# Identifying Key Principles and Commonalities in Digital Serious Game Design Frameworks: Scoping Review

**DOI:** 10.2196/54075

**Published:** 2025-03-05

**Authors:** Raluca Ionela Maxim, Joan Arnedo-Moreno

**Affiliations:** 1 Universitat Oberta de Catalunya Barcelona Spain

**Keywords:** entertainment game design frameworks, serious game design frameworks, design principles, empathic design thinking, artificial intelligence

## Abstract

**Background:**

Digital serious games (DSGs), designed for purposes beyond entertainment and consumed via electronic devices, have garnered attention for their potential to enhance learning and promote behavior change. Their effectiveness depends on the quality of their design. Frameworks for DSG design can guide the creation of engaging games tailored to objectives such as education, health, and social impact.

**Objective:**

This study aims to review, analyze, and synthesize the literature on digital entertainment game design frameworks and DSG design frameworks (DSGDFWs). The focus is on conceptual frameworks offering high-level guidance for the game creation process rather than component-specific tools. We explore how these frameworks can be applied to create impactful serious games in fields such as health care and education. Key goals include identifying design principles, commonalities, dependencies, gaps, and opportunities in the literature. Suggestions for future research include empathic design thinking, artificial intelligence integration, and iterative improvements. The findings culminate in a synthesized 4-phase design process, offering generic guidelines for designers and developers to create effective serious games that benefit society.

**Methods:**

A 2-phase methodology was used: a scoping literature review and cluster analysis. A targeted search across 7 databases (ACM, Scopus, Springer, IEEE, Elsevier, JMIR Publications, and SAGE) was conducted using PRISMA (Preferred Reporting Items for Systematic Reviews and Meta-Analyses) 2020 guidelines. Studies included academic or industry papers evaluating digital game design frameworks. Cluster analysis was applied to categorize the data, revealing trends and correlations among frameworks.

**Results:**

Of 987 papers initially identified, 25 (2.5%) met the inclusion criteria, with an additional 22 identified through snowballing, resulting in 47 papers. These papers presented 47 frameworks, including 16 (34%) digital entertainment game design frameworks and 31 (66%) DSGDFWs. Thematic analysis grouped frameworks into categories, identifying patterns and relationships between design elements. Commonalities, dependencies, and gaps were analyzed, highlighting opportunities for empathic design thinking and artificial intelligence applications. Key considerations in DSG design were identified and presented in a 4-phase design baseline with the outcome of a list of design guidelines that might, according to the literature, be applied to an end-to-end process of designing and building future innovative solutions.

**Conclusions:**

The main benefits of using DSGDFWs seem to be related to enhancing the effectiveness of serious games in achieving their intended objectives, such as learning, behavior change, and social impact. Limitations primarily seem to be related to constraints associated with the specific contexts in which the serious games are developed and used. Approaches in the future should be aimed at refining and adapting existing frameworks to different contexts and purposes, as well as exploring new frameworks that incorporate emerging technologies and design principles.

## Introduction

### Overview

Digital serious games (DSGs) have gained substantial attention and investigation in recent years, with a continuously expanding collection of scholarly works dedicated to this topic [1-3]. There are several definitions of serious games [4], but for this paper, the one proposed by Abt [5] is used: “we are concerned with serious games in the sense that these games have an explicit and carefully thought-out educational purpose and are not intended to be played primarily for amusement*.*” These games can be used for education, training, behavioral change, exergaming or therapeutic purposes, and other applications by using learning objectives in game mechanics to engage learners in an immersive learning experience [6,7].

Having a plan is the key when it comes to designing DSGs, particularly when the game is meant to serve an educational purpose beyond fun. Game design can be complex and cannot be improvised and that is why evaluating and improving design frameworks is essential. The purpose of such frameworks is to organize the design process into various phases or categories with specific guidelines and principles for each area, such as game design in education. This design flow is adjustable to different steps that involve evaluating the expected performance of a solution and identifying significant design challenges. The design process tackles each design issue and refines solutions based on information collected during the design flow, with each step informed by different guidelines and perspectives that impact the system. Therefore, the general goal of a framework design is to create solutions from preexisting components (analysis phase), to describe the critical decomposition problems, and to deliver a concrete example, not abstract (design and test phases) [8,9]. Because of their importance, there is a vast interest and amount of literature on DSGs with a variety of design frameworks that evolved to better serve experiences of users and learners. Frameworks and best practices exist to ensure that DSGs are designed appropriately [10-12].

The initial inspiration for the design of serious games derives from general fun or entertainment games. Things such as game mechanics (point systems and levels), player interaction (competition and collaboration), and visual design elements (appealing graphics and sound effects) are borrowed from games people play for enjoyment. Entertainment games prioritize fun, often featuring elaborate narratives and fantastical settings. However, serious games focus on achieving a specific learning objective. In essence, serious games borrow the engaging aspects of their entertainment counterparts to make learning more effective, as part of what Deterding et al [13] define as “the ludification of culture.” Serious games demonstrate the power of applying game design principles to achieve goals beyond pure entertainment. However, they are not simply educational resources dressed up with game mechanics. Instead, they are full-fledged games that integrate educational principles into their core design, as well as fostering motivation through game design approaches, making the learning experience not only effective but also engaging and enjoyable [14].

It is important to remark that serious games, despite their engaging nature, are designed to prioritize teaching or training players in specific skills or knowledge, encompassing fields such as science, arts, or even health promotion. Serious games leverage mechanics and technologies typically found in games to facilitate learning and potentially even influence behavior changes [15]. In contrast, entertainment games focus solely on providing players with enjoyable experiences. While the design considerations differ between these categories, both serious and entertainment games rely on engaging gameplay and immersive experiences to achieve success. In a purely digital context, video games have become a powerful force, encompassing both the realm of pure entertainment and the world of education and training [16]. Thus, as games become increasingly influential across various industries, understanding the design principles and frameworks behind both types, and their similarities and differences, becomes important [17].

The main objective of this research is a scoping review into the existing design frameworks in the literature that guide the creation of these diverse games. The focus lies on 2 distinct categories: digital entertainment game design frameworks (DEGDFWs) and DSG design frameworks (DSGDFWs). By grouping similar elements together, this analysis aimed to reveal commonalities and interdependencies between the 2 categories. This clustering process facilitated the identification of patterns, gaps, and opportunities within current design practices. This exploration serves 2 key purposes: first, to provide aspiring game designers with a clear understanding of existing frameworks, and second, to contribute valuable insights that can be used to develop more robust and versatile design tools for serious games in the future.

This paper is divided into several sections—first, establishing theoretical and conceptual foundations on digital games’ background and debriefing on entertainment and serious game design frameworks, next elaborating on the scoping review methods and qualitative or quantitative analyses conducted to evaluate relationships between game frameworks, then discussing key results found between serious and entertainment games, and finally synthesizing the results in a 4-phase design model for serious game development, which summarizes the main aspects identified in the literature review. The synthesis of findings into a 4-phase design visualization offers a structured approach to navigating the current serious game design space, fulfilling the need for accessible and informative resources in this domain. This baseline visualization aims to provide clarity to advance future applied initiatives and research by incorporating collective guidelines to drive the creation of serious games for scalable impact.

### Theoretical Background

DSGs are active and entertaining digital learning environments, accessible on computers and smartphones. Unlike pure entertainment games, their primary purpose is education and learning, “by combining with consistency, both serious aspects such as non-exhaustive and nonexclusive, teaching, learning, communication, or the information, with playful springs from the video game” as stated by Alvarez and Damien [18]. Through engaging gameplay and in-game challenges, players learn and develop practical concepts by applying their skills to overcome obstacles within the game. DSGs have been used in many different fields, such as health care, culture, training, business, and even to address social and political issues [4,17]. The variety of applications reflects the wide range of goals serious games can achieve, from raising awareness, to cultivating knowledge, to changing behavior [5,19].

Serious games can be in analog and digital format, whose differentiation lies in the platform they use to deliver the learning experience. Analog serious games typically involve physical components such as board games, card games, or role-playing activities. They offer tangible, hands-on experiences that promote collaboration, communication, and critical thinking skills. In contrast, DSGs are delivered through electronic platforms such as computers, consoles, or mobile devices. They often leverage multimedia elements such as graphics, sound, and interactivity to create immersive environments that enhance engagement and learning outcomes [7,20].

Creating impactful serious games involves additional challenges compared to entertainment games due to necessitating expertise spanning both game design fundamentals and instructional design strategies to guide outcomes. While serious games and entertainment games share certain surface-level characteristics such as interactivity, rules, challenges, and narrative elements, their fundamental purposes diverge [21,22]. Entertainment games primarily aim to provide captivating, rewarding experiences focused on having fun, emotional immersion, and intrinsic player motivation to progress further for self-actualization [23]. In contrast, serious games have explicit extrinsic goals beyond the gameplay itself, intending to educate users on new skills, catalyze developmental outcomes, or bring about behavioral changes—objectives tied to real-world usefulness. Overall, the research landscape on serious games applications and frameworks remains complex.

### Applications of DSGs

Although DSGs may be perceived as derived from entertainment games, their design necessitates additional expertise beyond traditional game design skills. Specifically, designing games for learning purposes demands proficiency in instructional design, student guidance, and assessment of learning outcomes [19,24]. Nevertheless, there is a connection between both types of games, which some researchers and designers explain as a combination of story, art, and software. Besides the game mechanics, player interaction, and visual design elements, the incorporation of educational principles into the game’s story is another significant characteristic of serious games. By integrating these principles into the story, the serious games transform learning from a passive activity into an active and engaging experience. Players become participants in the story, motivated by the narrative to explore, discover, and apply educational concepts within the immersive world of the game [25,26].

This connection enables the entertainment aspect of the game to effectively communicate the intended educational or training message. As a result, serious games provide a more engaging and effective experience than traditional methods of education and training, because they are more immersive and interactive [4,10]. For instance, in health care, serious games are used for patient education, training medical professionals, and rehabilitation [27]. In education, they are used to enhance student engagement and learning outcomes [28]. The military field uses serious games for training and simulation purposes. In the corporate world, they are used for employee training, leadership development, and team-building exercises [29]. In addition, serious games have also been used for public policy initiatives, such as disaster preparedness and environmental awareness campaigns [30].

In that regard, the use of serious games has been particularly effective in the health care sector, where complex medical concepts can be taught using games to both children and adults. Interactive games have also been proven to be an effective tool for educating young individuals about their health situations and medications [31,32]. The purpose of serious games’ design in the health care field is to support the gain of a general understanding of the previous work done, such as the use of games to reduce knowledge gaps on various topics, which can aid in improving the effectiveness of game development [33]. For instance, a recent serious game has been designed to make the medical hematology topic more engaging for health care students, encouraging them to analyze blood tests to provide better care for their patients. This game-based approach has proved to enhance learners’ knowledge of hematology, providing an effective tool for education and skills development [34].

All those mentioned fields underline the usefulness of serious games, whose main purpose is to teach learners different topics in joyful and interactive experiences. An important component of serious game design is represented by the learning theories mixed with hedonic elements. Learning theories used in serious games can vary and are dependent on the game’s specific objectives. For instance, some serious games may be based on constructivism, which highlights the learner’s role in constructing knowledge through active exploration and discovery [35]. Alternatively, some games may be based on behaviorism, which concentrates on the observable behavior of the learner and the application of rewards and punishments to influence behavior. Moreover, other games may use cognitive behavioral theories, which focus on the relationship among thoughts, emotions, and behaviors [36].

To create a great DSG, designers need to turn goals into a clear picture of what the game will be. This involves understanding what kind of learning experience they want to create. The team then asks a series of questions to define the game’s mechanics: what specific skills or knowledge will it teach, what emotions and feelings should players have while learning, and how will the game itself (objectives, rules, and story) achieve this. Designing DSGs requires considering various aspects of playful learning, including how people think (cognitive), feel (affective), and are motivated (motivational), alongside social and cultural factors (sociocultural). The learners’ emotional engagement is powerful and strongly correlated to their deeper cognitive effort [26,37,38].

In essence, mixing all hedonic and pragmatic elements is essential for the development of various serious games solutions. However, their effective and successful creation should start from a formal design process and methodology that provide the essential guidance for development teams. However, designing serious games is not a straightforward process, as there is no single framework or theory that can be applied to every type of serious game.

### Game Design Frameworks

Generally speaking, design frameworks are systematic approaches used to design and develop products. They provide a structure for designers and developers to follow, helping to ensure that the final designed product meets the desired objectives and user needs. The concept of a framework is defined as a skeleton structure of theories and guiding principles that shape and organize the components of a phenomenon or identified problem [39,40]. However, creating a design framework that satisfies the complexity of different approaches remains a challenge for designers and developers [41].

There are different adapted approaches applied to both entertainment and serious games, starting with their main focus and goals. Entertainment games target the creation of engaging and immersive gameplay, using components such as game mechanics, esthetic experiences in gameplay, and reward systems to create a fun and enjoyable experience for players with the goal of commercial success on one hand [42-44]. On the other hand, serious games are designed with the educational purpose of creating effective and motivating learning experiences, even though not everyone agrees. One study argues that serious games are more effective for learning and retention than traditional teaching but not more motivating than conventional methods [45]. Other studies state that serious games cultivate greater learner motivation through incorporating interactivity, competition, social connections, achievement-based progression systems, and freeform experimentation appeal unavailable in conventional coursework [46].

Serious games often incorporate elements such as instructional design that turns education into game-based learning. These elements are tailored to specific learning objectives and curriculum standards, while also considering both accessibility and user-centered design to ensure ease of use and usefulness for diverse users. The process of education can be viewed as a game-like journey with challenges and goals. Motivation is a key aspect of engagement in both education and games, leading to an interest in applying motivational strategies from games to education. However, a challenge in doing so is creating a game scenario that feels natural and not artificial. Game designers emphasize that the game is a tool for achieving an experience, rather than the experience itself. Therefore, it is important to understand game design principles when applying game elements to education [47].

In addition, most existing serious games frameworks are designed for specific types of educational games. This is due to the diversity of serious games and the various learning paradigms and contexts in which they are used. Existing design frameworks for serious games also differ in their objectives. Some focus more on the design elements of serious games, while others emphasize the design and development process. Some frameworks cover both design elements and process, while others are more concerned with the theoretical foundations of game-based learning [39,48]. Therefore, when starting a new serious game development plan, the design team must make a decision on a specific framework to work with [48-50].

### Designing Digital Entertainment Games

Digital entertainment games (DEGs) are typically designed for the purpose of providing enjoyment to players. They often involve fictional worlds, characters, and storylines and can take the form of action games, adventure games, puzzle games, and more. They can be played on various platforms and interfaces, including console, PC, or mobile [6]. The game industry allows for a wide range of options in game design and creation, with practical considerations such as time constraints, financial budgeting, and available personnel often dictating the process. While there is no one-size-fits-all formula for creating a game, certain practices have emerged over time through trial and error and successful results. These practices include the use of frameworks in design, but information about these frameworks is inconsistent from one proposal to another [51].

Inconsistencies in game design frameworks can arise from various factors, including the lack of standardized guidelines or universally accepted best practices, the rapidly evolving nature of the industry, diverse design goals and objectives, and varying target audiences. It is important to note that these inconsistencies are not necessarily negative, as they can offer a diverse range of perspectives and approaches to game design, enabling designers to select and adapt frameworks that best align with their specific needs and design goals. These inconsistencies also highlight the dynamic and ever-evolving nature of the game design field, which constantly adjusts to changing technologies, player preferences, and design trends. For instance, these frameworks may differ in their focuses, such as player motivations (Bartle taxonomy) [43], types of fun (Four Keys to Fun) [52], player personalities (Engines of Play) [53], or player involvement (Player Involvement Model) [54]. They may also vary in their scopes, with some frameworks concentrating on specific aspects of gameplay, such as mechanics, dynamics, and aesthetics [55], while others adopt a more holistic approach that considers multiple elements, such as formal, dramatic, and dynamic elements, esthetics, and story (Layered Tetrad) [56].

Entertainment game frameworks can influence educational serious games by providing structures for creating engaging and interactive gameplay, adapting well-designed user interfaces and navigation systems, and incorporating well-developed storytelling and narrative elements to create immersive and engaging educational experiences. In addition, entertainment games have a wide range of mechanics and gameplay elements that can be used to create educational games tailored to different subjects and learning styles [57].

### Designing DSGs

DSGs are designed to provide a learning experience that balances both educational and fun aspects equally. Motivation is a very important factor in their development, as it is essential for engagement in education. Incorporating motivational strategies from DEG has become a growing interest in education [53]. Collaboration and immersion are important aspects that designers should consider when creating DSGs. They allow players to learn and experience together in a digital or virtual environment [58,59]. Despite their effectiveness, for example, in the computer science area, designing them requires additional effort during the design process to cater to learners with varying abilities and needs [60].

In fact, education can be seen as a journey toward acquiring knowledge, similar to the experience of playing a game. Tests and practical work can be thought of as challenges, and the desired learning outcomes can be thought of as the goals to be achieved. The purpose of this journey is to change the student or learner through innovative experiences such as some DSG with virtual reality or augmented reality that combines multisensory experiences [61]. Game designers often argue that the game itself is not the experience but rather a tool for achieving an experience. Therefore, it is essential to have the know-how of game design when creating game scenarios to avoid creating a forced or artificial game environment [62]. A typical method of validating serious games is analyzing individual game mechanics to understand their impact on player behavior and the overall gaming experience [63].

Though there is not much research on how individual aspects of game design contribute to the effectiveness of serious games, it is important to thoroughly test individual aspects of their design to ensure their effects. In addition, their purpose and perceived value may also play a role in its success, as players may have a more positive attitude toward a game if they consider it to be beneficial. Another important aspect for more useful and usable serious game design would be the incorporation of empathic design principles by involving lecturers and learners in the design process [64].

### Learning Theories for DSGs

Effective DSG design is grounded in an understanding of various learning theories that can be applied in game development [65]. Constructivism, for example, emphasizes the learner’s active role in constructing their understanding of the world through open-ended problem-solving tasks, experiences, and digital interactions [66]. Cognitive load theory is another important learning theory applied in serious game design, which posits that excessive cognitive load can interfere with learning [66]. To minimize extraneous cognitive load, immersive game environments that use multimedia and visual aids to support learning are used.

Social learning theory is yet another important learning theory applied in serious game design. This theory emphasizes learning through observation and imitation of others, and collaborative and multiplayer game environments that allow learners to work together and learn from one another are often used to implement this theory [67]. Self-determination theory, which supports the fact that learners are more motivated and engaged when they have autonomy and control over their learning experiences, is also applied in serious game design [68]. Personalization and adaptive game mechanics that allow learners to set their own goals and progress at their own pace are used to support self-directed learning.

In addition, behaviorism is a learning theory that underlines the role of environmental factors in shaping behavior, and it is applied in serious game design by providing immediate feedback to players and rewarding or punishing certain behaviors [69]. Goal-oriented instruction is another learning theory that focuses on the achievement of specific goals or objectives in a learning environment, which aligns well with the objectives of educational games [35]. Problem-based learning is another interesting learning theory that emphasizes solving real-world problems to learn, which aligns well with the interactive and hands-on nature of games [68]. Video games can be valuable educational tools when effectively integrated with learning theories. It is important to examine both the advantages and obstacles in aiding learning. This includes exploring strategies for integrating video games into educational settings and understanding their impact on student engagement, motivation, and cognitive development [70].

Overall, effective serious game design needs to be rooted in an understanding of various learning theories that inform game development. Constructivism highlights the learner’s active role in constructing an understanding through problem-solving tasks and digital interactions, while cognitive load theory addresses the impact of excessive cognitive load on learning. Social learning theory emphasizes learning through observation and collaboration, while self-determination theory supports learner motivation through autonomy. Behaviorism is applied through feedback and reinforcement, while goal-oriented instruction and problem-based learning align with the objectives of educational games. When integrated effectively, serious games have the potential to be valuable educational tools, impacting student engagement, motivation, and cognitive development.

## Methods

A comprehensive literature review was conducted to establish a foundational understanding as a first phase, followed by a cluster analysis to categorize data into meaningful groups and identify trends and correlations within the design frameworks as a second phase in this study.

### Literature Review

As a first step, a comprehensive literature review was conducted to gather existing knowledge and insights on the topic, laying the foundation for the subsequent cluster analysis.

While there are frameworks for other game formats such as hands-on analog play, instructor-guided play, or e-books, the focus of this study is to specifically explore frameworks tailored to the unique characteristics of DEGs and DSGs. The widespread integration of digital games and gamification into education highlights the importance of grasping the foundations and design principles essential for creating impactful game-based learning encounters [25]. This paper is analyzing frameworks that can be used in digital games but not only necessarily for digital games. Specifically, the focus of the review is on both DEG and DSG design frameworks, including their various design elements and principles. The study aims to answer several questions, such as which digital game design frameworks are available in the literature and what their main features are and how entertainment game design principles might influence serious game design frameworks.

The identification and reporting process of the studies included in the review were guided by the PRISMA (Preferred Reporting Items for Systematic Reviews and Meta-Analyses) checklist [71]. The PRISMA statement with the completed checklist provided in Multimedia Appendix 1, allowed to better organize the reporting of the systematic review with some steps: database search of the papers based on all desired search terms, duplicate papers removal, screening of titles and abstracts and excluding the papers that are not relevant for our research questions, full-text screening to assess the eligibility and exclude again the papers that did not contain prototypes with needed design details, and final mention of the included studies. No preregistration or protocol was completed before the literature search.

This literature review aims to analyze the details related to digital entertainment and serious games design frameworks using a systematic mapping approach. The papers selected were searched on 6 electronic databases: ACM, Scopus, Springer, IEEE, Elsevier, JMIR Publications, and SAGE. The search keywords included “game design frameworks,” “serious game design frameworks,” “game design guidelines,” and “serious game design guidelines.”

The search strategy, presented in Multimedia Appendix 2, focused on two key aspects: (1) serious games and (2) entertainment games (the search keywords used were “design” OR “frameworks” OR “guidelines”). Papers that used similar but not these exact terms were identified during the abstract reading phase, ensuring a comprehensive capture of relevant literature. To ensure the relevance and quality of the selected works, inclusion and exclusion criteria were rigorously applied. The inclusion criteria encompassed academic works (not papers only) explicitly identified “as a framework” in peer-reviewed literature. This research seeks academic publications from the years 2015 to 2023, which outline established frameworks and recommendations for structuring the design and development of games. The focus is on understanding how these frameworks provide a systematic approach to game creation, while guidelines offer best practices for developers. The analysis also examines key design elements common to successful games and identify the intended learning outcomes associated with them.

Conversely, the exclusion criteria ruled out papers that mention game design without detailed phase definitions, contain models as simplified process representations, or reference serious games without defining framework details. Moreover, the exclusion criteria eliminate the works that never explicitly were identified as “frameworks” when referenced by other authors in the peer-reviewed literature. The study is open to non–peer-reviewed works but only if peer-reviewed works explicitly consider them frameworks.

Duplicate papers were removed, and a manual selection process was used to narrow down the number of papers. The titles and abstracts of the papers were carefully examined to determine whether they met the inclusion criteria. After the initial manual selection, the selected papers were read in detail to ensure they met the inclusion criteria, and further elimination of irrelevant papers was done to narrow down the papers to those most relevant to the research questions.

Moreover, a snowball process was performed in addition to the initial search strategy to ensure a comprehensive scoping review [72]. This process involved identifying and analyzing papers that were referenced by the previously identified design frameworks. It should be noted that this snowball process was limited to papers that referenced design frameworks, even if they were published before 2015. This was done to ensure that all relevant papers were included in the review, regardless of their publication date, by revealing additional key works that are influential in the field and leading to a more robust understanding of the topic. This process allowed the exploration of the relationship between different design frameworks and identification of any additional design guidelines or good practices that were not captured in the initial search.

The synthesis of the selected studies was carried out using a thematic analysis approach, where the design frameworks identified in the reviewed papers were first classified into 2 broad categories: DEGDFWs and DSGDFWs. Each framework was assessed for its relevance to the characteristics of digital entertainment and serious games, and the subsequent cluster analysis for commonalities and differences was applied in the dedicated Results section of this paper.

### Clustering

Following the comprehensive literature review, which provided a foundational understanding of the existing research landscape, we proceeded with a more granular examination through cluster analysis. In this second phase of the research journey, we applied clustering techniques to categorize the data into meaningful groups, allowing us to explore specific trends and correlations that were not immediately apparent in the earlier stages.

#### General Clustering

To categorize the frameworks, clustering through thematic analysis coding was done with the MAXQDA (VERBI GmbH) software for qualitative data analysis. Clustering refers to the task of grouping a set of objects in such a way that objects in the same group are more similar to each other than to those in other groups. The frameworks were clustered into distinct conceptual themes based on their focus areas. This process involved coding the frameworks’ key elements, analyzing the relationships between codes, and ultimately grouping them into broader themes that form the final clusters.

The clustering process, conducted collaboratively by 2 coders, involved several steps. First, each framework was reviewed to create initial codes based on its primary focus and objectives (game mechanics, learning objectives, etc). An inductive approach was used in this process, where codes and themes were identified directly from the data based on observed patterns rather than predefined categories. This method allowed new insights to emerge and themes to be developed that accurately reflected the frameworks’ focus area [73]. By using MAXQDA, the coders were able to create, assign, and visualize codes related to the primary focus areas of each framework. This software enabled efficient identification of patterns and relationships between the codes, helping to reveal commonalities and cooccurrences across the frameworks.

After coding, commonalities and relationships (frequency of codes and their cooccurrences) between frameworks were identified to develop higher-level themes. For example, the codes “learning objectives” and “educational content” were grouped into the theme “learning-oriented frameworks,” while “gameplay elements” and “game mechanics” were grouped under “game design–oriented frameworks” (the full list of the identified codes is discussed in the Clustering Results section). The coders largely agreed on the final clusters, which are described in Multimedia Appendix 3, for distribution of the frameworks into each corresponding cluster.

The discussions among coders were structured around collaborative analysis sessions, where they reviewed the initial codes and debated their meanings and implications. This iterative process involved each coder presenting their interpretations and justifications for specific codes, allowing for a comprehensive examination of the frameworks in question. During these discussions, the coders identified common themes by highlighting overlaps and distinctions among the codes. They focused on the context and nuances of each code, fostering a dialogue that encouraged critical thinking and reflection on the broader implications of their findings. Moreover, the fit of clustering (intercoder agreement) was calculated using Cohen κ [74], which accounts for agreement by chance of coders. The Cohen κ value was 0.85, indicating a high level of agreement between the coders. This suggests that the frameworks were well categorized within the identified clusters.

#### Dependencies and Similarities Clustering

To analyze the potential dependencies and similarities between DSGDFWs and DEGDFWs, a comprehensive clustering procedure was also used. This process combined qualitative coding and quantitative network analysis using MAXQDA and R (R Foundation for Statistical Computing) to uncover the frameworks’ conceptual links and dependencies.

In the first phase, data on framework dependencies were coded in MAXQDA, and a code cooccurrence matrix was created to quantify conceptual associations. For example, the “activist-casual framework” was identified as depending on the “Bartle taxonomy” to create games focused on delivering a customized player experience based on performance levels. In MAXQDA, qualitative coding was applied to these data. Codes such as “custom player experience” and “performance level dependence” were created and applied to segments describing these dependencies. Furthermore, in the second phase, a code cooccurrence matrix was derived in MAXQDA. For instance, the matrix indicates that “custom player experience” and “performance level dependence” frequently cooccur.

This matrix was then exported to the statistical software R, where a similarity matrix was derived to reflect the degree of relatedness between frameworks. For example, frameworks “activist-casual framework” and “Bartle taxonomy,” sharing high similarity scores, were shown to be closely related. A distance matrix was derived from this similarity matrix, indicating how similar or different the frameworks were in terms of their theoretical dependencies. Clustering algorithms were applied to group frameworks into clusters based on these similarities. For example, let us presume that the frameworks such as “activist-casual framework” and “Bartle taxonomy” were assigned to clusters based on these analyses. In the third phase, a concept network graph was generated in R to visualize these clusters and the dependency flows between frameworks.

The final part of the clustering procedure involved interpreting the clusters to define overarching principles. Specifically, once the clustering algorithms had grouped the frameworks based on their conceptual dependencies, each cluster was analyzed to identify the shared characteristics and design philosophies of the frameworks within it. Consequently, these insights were used to associate each cluster with a specific principle. For instance, the principle of enjoyable education emerged from the analysis of a cluster where frameworks commonly emphasized balancing fun and educational content. This process ensured that each principle was grounded in the actual design practices and theoretical foundations reflected in the clusters.

Overall, by organizing these codes and analyzing their frequencies and relationships, we could highlight framework domains or categories and assess commonalities and differences across them. This enabled us to uncover conceptual clusters across the game design frameworks. The following section highlights the results of the clustering analysis.

### Risk of Bias Assessment

To ensure a reliable and accurate analysis, the management of risk of bias was conducted using the MAXQDA [75] tool for qualitative data analysis and synthesis. This was done to refine and organize the relevant information. In addition to the tool, a manual assessment was performed by 2 reviewers to further verify the findings. This double approach was taken to ensure that the analysis was as thorough and accurate as possible.

## Results

The results are structured into 2 main parts: findings from the literature review, which provide an overview of existing game design frameworks, and insights from the clustering analysis, which categorize and highlight patterns and relationships within the frameworks.

### Literature Review Results

This review focused specifically on analyzing high-level conceptual frameworks that provide overarching guidance on the entire game design and development process and not narrower design lenses. The goal was to elucidate broader theoretical scaffolding around core phases, methodologies, needs analysis factors, and evaluative pillars that inform practice foundations. Hence, the review concentrated on understanding process-oriented knowledge generalizable across use cases rather than tactical formulas for crafting individual interactions. In summary, the scope covered conceptual frameworks directing holistic developmental sequences rather than granular tools optimizing precise design decisions.

**Figure 1:**
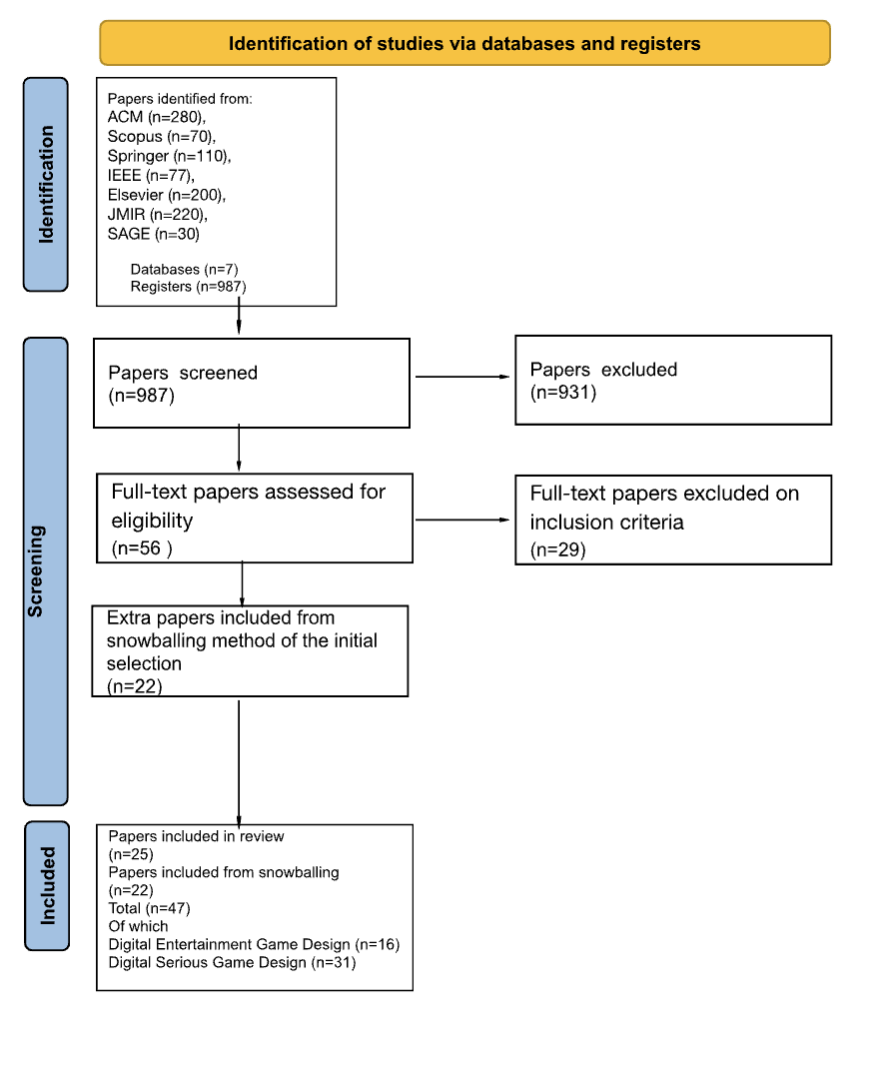
PRISMA (Preferred Reporting Items for Systematic Reviews and Meta-Analyses) diagram.

Figure 1 presents a PRISMA 2020 diagram outlining the study identification process. The research strategy initially identified 987 papers, of which 54 (5.5%) were selected based on the inclusion and exclusion criteria. The search parameters yielded the following number of results for each database: ACM (280/987, 28.4%), Scopus (70/987, 7.1%), Springer (110/987, 11.1%), IEEE (77/987, 7.8%), Elsevier (200/987, 20.3%), JMIR Publications (220/987, 22.3%), and SAGE (30/987, 3%).

Following a second review, 25 papers were chosen for full reading and synthesis, focusing on design framework components. To ensure a comprehensive review, a snowballing method was applied in addition to the initial search. This involved analyzing papers cited by the identified design frameworks, including an additional 22 papers.

Ultimately, 2 types of frameworks were identified from a total of 47 papers: 25 (53%) from the initial selection and 22 (47%) from the snowballing process. The study synthesized 47 frameworks overall, with 16 (34%) related to DEGDFW (Table 1) and 31 (66%) related to DSGDFW (Table 2). The details of the frameworks can be found in Multimedia Appendices 4 and 5.

**Table 1:** Summary of the reviewed digital entertainment game design frameworks (DEGDFWs).

ID code	DEGDFW
EG^a^1	The Bartle taxonomy [43]
EG2	The Four Keys to Fun [52]
EG3	The Engines of Play [53]
EG4	The Player Involvement Model [54]
EG5	MDA (Mechanics, Dynamics, Aesthetics) [55]
EG6	The Layered Tetrad [56]
EG7	Design, Dynamics, Experience (DDE) [76]
EG8	Elemental Tetrad [77]
EG9	MTDA+N Narratives framework [78]
EG10	Integrated Framework for Game Design [79]
EG11	The 5-part Model [80]
EG12	Risk and Reward Model, (difficulty balancing) [44]
EG13	Game Design Patterns Model [81]
EG14	Game design framework [23]
EG15	Game design workshop [82]
EG16	Game Element and Mechanic framework [83]

^a^EG: entertainment game.

**Table 2:** Summary of the reviewed digital serious game design frameworks (DSGDFWs).

ID code	DSGDFW
SG^a^1	Activist-Casual framework [84]
SG2	Educational Games Design framework [21]
SG3	Design, Play, Experience framework [85]
SG4	Conceptual framework [86]
SG5	Four Dimensional Framework [87]
SG6	Triadic game design evaluation framework [88]
SG7	RETAIN framework [89]
SG8	Computational Puzzle Design framework [90]
SG9	Serious educational games framework [91]
SG10	FRACH framework [58]
SG11	Augmented intelligence framework [92]
SG12	Lu Lu framework [93]
SG13	Learning Mechanics-Game Mechanics [94]
SG14	Digital Game–Based Learning–Instructional Design Model [95]
SG15	Immersive Educational Games Model [96]
SG16	Design, Dynamics, Experience (DDE) framework [76]
SG17	MECONESIS methodology [97]
SG18	Framework with game design, learning content modeling, and pedagogy [21]
SG19	Methodology based on graphic notation and interactive narrative [98]
SG20	Methodology based on cognitive behavioral techniques [99]
SG21	EMERGO methodology [100]
SG22	ATMSG framework based on Activity Theory [22]
SG23	Methodology based on problem-based learning [101]
SG24	Six-dimensional framework in a reviewed paper [59]
SG25	Baseline content-centric framework [102]
SG26	GAMED framework [103]
SG27	User-centered design methodology [104]
SG28	Collaborative Learning Game framework [105]
SG29	Conceptual Model for Serious Games [106]
SG30	Adaptive Learning Game Design Model [107]
SG31	Intervention Mapping Framework [108]

^a^SG: serious game.

Following the literature review, the Clustering Results section presents the cluster analysis conducted to further explore and categorize the identified frameworks, providing additional insights into their relationships and design elements.

### Clustering Results

Building on the findings from the literature review, the cluster analysis delves deeper into the relationships and patterns between DSGDFWs and DEGDFWs, offering a detailed examination of their commonalities, dependencies, and key design principles.

#### Purpose of Game Design Frameworks in Practice

Frameworks are essential tools that help designers and developers create effective and engaging educational games. These frameworks come in different types (Textbox 1), each with its own purpose, as shown in Multimedia Appendix 3.

**Types of frameworks.**

- Learning-oriented frameworks: these frameworks focus on the integration of educational content and learning objectives into the game design process. Examples include the Conceptual Model for Serious Games, the Dimensional Framework, the Educational Games Design Framework, and the Serious Educational Games Framework.
- Game design–oriented frameworks: these frameworks focus on the game mechanics, game design, and gameplay elements that are necessary to create engaging and effective educational games. Examples include the Learning Mechanics—Game Mechanics model; the Immersive Educational Games Model; the Design, Dynamics, Experience framework; the Computational Puzzle Design framework; and the Collaborative Learning Game framework.
- User-centered frameworks: these frameworks focus on understanding the needs and preferences of the users and providing a personalized experience for them. Examples include user-centered design and the mild cognitive impairment game therapy experience (MCI-GaTE) frameworks.
- Game development–oriented frameworks: these frameworks provide a structured approach for overcoming the complexity of game development and provide a guide for the development process. Examples include the GAMED methodology and the MCI-GaTE frameworks.
- Immersivity and collaboration-oriented frameworks: these frameworks focus on the importance of immersivity and collaboration when promoting group learning. Examples include the FRACH and the serious educational games frameworks.
- Mobile and ubiquitous learning–oriented frameworks: these frameworks focus on the various components of mobile game–based learning to provide learners with a mobile or ubiquitous learning experience. Examples include the six-dimensional framework.

#### Commonalities in DEGDFWs

Despite their differences in scope and focus, many of these game design frameworks share some commonalities (Textbox 2).

**Commonalities among entertainment game design (DEG-C) frameworks.**

1. Emphasis on player experience (DEG-C1): the player experience is a key focus in many game design frameworks. Creating an enjoyable and engaging experience for players is seen as essential in keeping them motivated and invested in the game. By prioritizing player experience, designers can create games that are not only educational but also enjoyable to play.
2. Consideration of game mechanics (DEG-C2): game mechanics are essential in game design frameworks, as they play a significant role in creating a fun and challenging game. The various game mechanics used in a game are useful in determining the level of challenge, reward, and engagement the game provides. By emphasizing game mechanics, designers can create games that are not only fun to play but also educational and informative.
3. Interconnectivity of game components (DEG-C3): many entertainment game design frameworks acknowledge the interconnectivity of game components such as mechanics, esthetics, story, and technology. The game mechanics, esthetics, and story all work together to create an immersive and cohesive game that can transport players to another world. By recognizing this interconnectivity, designers can create games that provide a seamless and enjoyable experience for players.
4. Importance of play testing (DEG-C4): several entertainment game design frameworks emphasize the importance of play testing in refining game design and improving the player experience. Play testing allows designers to identify areas for improvement in game mechanics, difficulty, and engagement. By iterating on the game design based on feedback from play testing, designers can create games that are more enjoyable and effective.
5. Need for iteration (DEG-C5): iterating on game design is recommended in many game design frameworks as a means to improve the player experience. By adjusting difficulty, refining game mechanics, and play testing, designers can create games that are more engaging and effective. Iteration is also essential in keeping up with technological advancements and changing user preferences.
6. Focus on understanding player motivations (DEG-C6): some game design frameworks suggest that understanding player motivations and personalities can help tailor game design to appeal to specific player types. By identifying what motivates players to play, designers can create games that are more engaging and enjoyable. Understanding player motivations also helps designers create games that meet specific learning objectives and goals.
7. Acknowledgment of the importance of storytelling (DEG-C7): it is emphasized in some game design frameworks as a means of creating an immersive game world and engaging players. By incorporating a compelling story into the game, designers can transport players to another world and provide a more enjoyable and engaging experience. Storytelling also helps designers create games that are more educational and informative by conveying information in a narrative format.

#### Commonalities in DSGDFWs

The analyzed serious game design frameworks underline some commonalities that are important because they provide a foundation for creating effective serious games (Textbox 3).

**Commonalities among serious game design (DSG-C) frameworks.**

1. Educational content with gameplay mechanics (DSG-C1): by incorporating educational content into games, designers can create immersive and engaging learning experiences for players. Serious games have the potential to enhance learning outcomes by using gameplay mechanics to motivate and engage learners in educational content.
2. Instructional design principles (DSG-C2): serious game development follows a systematic and structured approach that includes instructional design principles and phases. The process involves defining learning objectives, analyzing the target audience, designing the game mechanics, developing the game, testing it, and evaluating its effectiveness.
3. Immersive gameplay elements (DSG-C3): immersive elements help designers to create a more engaging and compelling learning experience for players. Immersive elements, such as virtual reality, audio and visual effects, and realistic scenarios, help create a sense of presence and realism that can enhance the learning experience.
4. Player experience and feedback (DSG-C4): designers need to consider the player’s experience throughout the game development process to ensure that the game is engaging and effective. Collecting player feedback helps identify areas for improvement, which can be addressed to improve the game’s usability, ease of use, functionality, and effectiveness.
5. Pedagogy and learning content modeling (DSG-C5): the integration of these 2 elements helps ensure that the game is aligned with the desired learning outcomes. Pedagogy helps designers create effective instructional strategies, while learning content modeling helps them organize and structure the educational content.
6. Cognitive and psychological aspects of players (DSG-C6): designers need to consider the cognitive and psychological aspects of the player, such as attention, memory, emotion, and motivation, to create an engaging and emotional gameplay experience. By incorporating these elements into the game design, designers can create a more personalized and effective learning experience for the player.
7. Collaboration and communication (DSG-C7): the emphasis on collaboration and communication among team members is essential to facilitate the exchange of ideas and artifacts. This collaborative approach helps ensure that the game is designed effectively, aligns with the learning objectives, and meets the needs of the learners.

#### Dependencies Between DSGDFWs and DEGDFWs

DEGs are popular because they provide players with enjoyable and engaging experiences that allow them to escape from reality and immerse themselves in a different world. DEGs are designed to be fun and pleasing, with challenging gameplay mechanics and compelling storylines that keep players coming back for more. Therefore, DSGs have extended from DEGs because they recognize the potential of games as a tool for education, training, and other serious applications.

There are several dependencies that exist between DSGDFWs and DEGDFWs, as both types of games share some commonalities. For instance, both types of digital game design frameworks emphasize the importance of player experience and game mechanics. In DEGs, game mechanics are important in creating a winsome and challenging game, while in DSGs, game mechanics are essential in delivering an immersive and attractive learning experience. Similarly, both types of games acknowledge the importance of play testing and iteration to refine game design and improve the end-to-end player experience.

Another common characteristic between DSGDFWs and DEGDFWs is the emphasis on understanding player motivations. In DEGs, understanding player motivations can help tailor game design to appeal to specific player types, while in DSGs, they are pillars to create games that meet specific learning objectives and outcomes.

Storytelling is also an important design element identified in both DSGs and DEGs, as it supports an immersive game world and captivation of players. In DEGs, storytelling is used to provide a more compelling experience, while in DSGs, it underlines the conveyance of educational content in a narrative format. Overall, both serious and entertainment games design frameworks share several commonalities and dependencies, highlighting the importance of considering both types of games when designing effective and engaging games.

**Figure 2:**
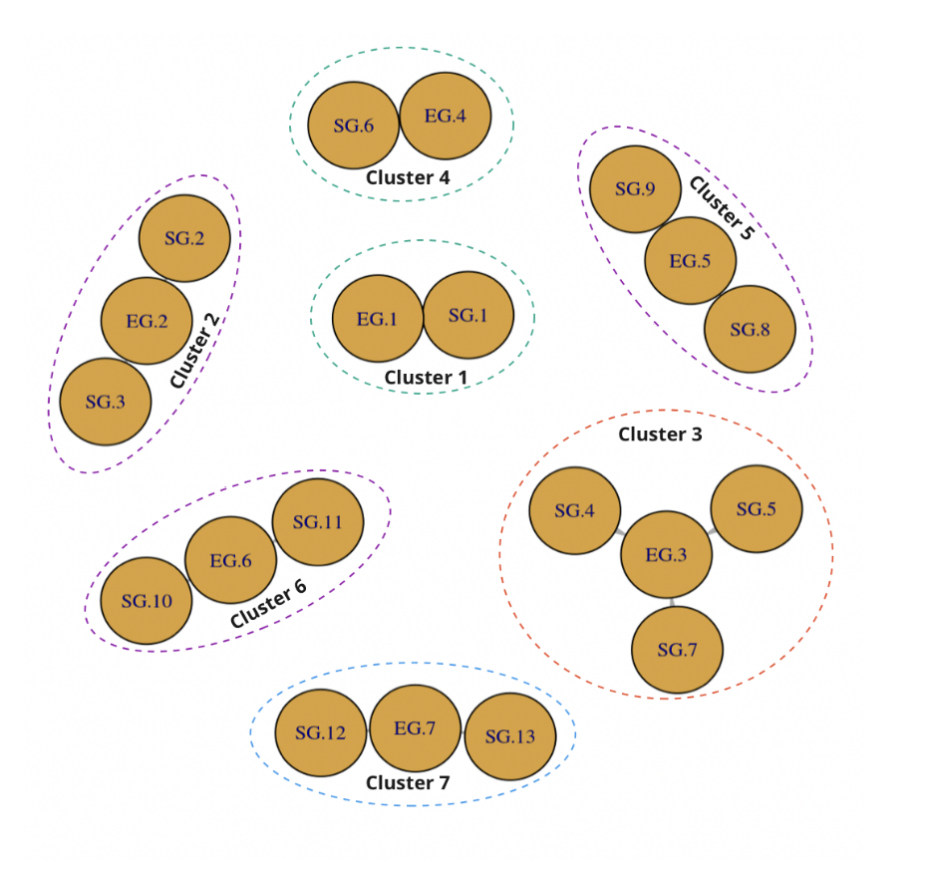
Graph with clusters on dependencies between SG.1-13 and DG.1-7 coded design frameworks.

The scoping review identified 2 main types of frameworks from the 47 papers analyzed: 16 (34%) DEGDFWs (Table 1) and 31 (66%) DSGDFWs (Table 2). Of the 47 frameworks, 25 (53%) were initially selected, and an additional 22 (47%) were gathered through snowballing techniques. Figure 2 illustrates a graph showing the dependencies between 13 DSGDFWs and 7 DEGDFWs, organized into 7 clusters. These specific frameworks were chosen based on their prominence and relevance in the literature, as they demonstrated significant interdependencies and were most representative of the key principles identified in the study. Either the remaining frameworks did not show significant relationships, often forming isolated or single-node clusters, or their inclusion did not contribute substantially to the overarching themes and trends we aimed to analyze.

A subset of the design frameworks discussed in the Literature Review section were clustered with the associated ID code. These ID codes correspond to the papers listed in Tables 1 and 2, where each paper is assigned a unique identifier for easier reference. The selected frameworks for clustering were chosen based on their relevance to the research objectives, while other papers either formed single-node clusters or did not yield significant patterns in this analysis. The clusters are visually represented in Figure 2, allowing for clear identification and comparison (Textbox 4).

**Clusters of design frameworks for serious games (SGs) and entertainment games (EGs).**

1. Cluster 1: the first dependency shown in this graph is the Activist-Casual framework (SG.1), which depends on the Bartle taxonomy (EG.1) to create games that focus on delivering a custom experience to players based on performance level.
2. Cluster 2: the Serious Educational Games (SG.2) and Design, Play, and Experience (SG.3) frameworks both depend on the Four Keys to Fun (EG.2) framework to create games that are fun and engaging while also providing educational content.
3. Cluster 3: the Conceptual Model (SG.4) and Four Dimension (SG.5) frameworks both rely on the Engines of Play (EG.3) framework to create games that are challenging and immersive while also incorporating compelling storylines. The RETAIN framework (SG.7) also relies on the Engines of Play framework (EG.3) to create games that are designed to be played repeatedly and encourage players to a continuous learning experience.
4. Cluster 4: the Triadic Game Design (SG.6) framework depends on the Player Involvement Model (EG.4) to create games that are tailored to the individual learning styles and preferences of each player.
5. Cluster 5: the Computational Puzzle Design (SG.8) and SG-LMI (SG.9) frameworks both depend on the Mechanics, Dynamics, and Aesthetics (EG.5) framework, which prioritizes the player’s experience and journey throughout the game. SG.8 uses the EG.5 framework to create mechanics that facilitate problem-solving through programming, while SG.9 uses it to create an immersive and engaging game experience that supports effective learning outcomes.
6. Cluster 6: the FRACH Serious Game (SG.10) and Augmented Intelligence (SG.11) frameworks both depend on the Layered Tetrad Model (EG.6) to create games that are designed to be immersive and engaging while also incorporating elements of augmented reality and artificial intelligence. SG.10 applies the EG.6 model to incorporate game mechanics and learning objectives, while SG.11 uses the EG.6 model to create an augmented intelligence system that adapts to the player’s learning needs.
7. Cluster 7: the LU LU framework (SG.12) and mild cognitive impairment game therapy experience framework (SG.13) both depend on the Design, Dynamics, and Experience (EG.7) framework to create games that are designed to be fun and engaging while also incorporating educational content and promoting positive behaviors.

Principles (PR1-6) derived from the clusters of dependencies between frameworks (Textbox 5).

Overall, while DSGDFWs are focused on delivering educational content to players, they rely heavily on DEGDFWs to ensure that the games they create are enjoyable and engaging for players. DEGDFWs provide DSG designers with a set of tools and techniques that they can use to make their games more immersive and impactful. By leveraging the commonalities and dependencies between DSGDFWs and DEGDFWs, designers can create games that are both educational and entertaining, which is essential to attracting and retaining players. As the field of DSG continues to evolve, it will be interesting to see how designers continue to leverage DEGDFWs to create impactful and meaningful experiences for players.

**Principles (PR) derived from the clusters.**

- Challenge tailoring (PR1): design gameplay mechanics and challenges that are tailored to the abilities and progress of players, providing an engaging experience. By adjusting the difficulty level or introducing adaptive elements, players are motivated to push their boundaries and feel a sense of accomplishment as they overcome challenges suitable for their skill level. Cluster 1 focuses on creating games that offer a customized experience based on performance levels. This aligns with the principle of challenge tailoring, which emphasizes designing gameplay mechanics and challenges tailored to players’ abilities and progress.
- Enjoyable education (PR2): balancing enjoyment and educational content is crucial. Games should provide enjoyable experiences that captivate players’ attention and interest while effectively delivering educational material. This can be achieved through incorporating interactive learning elements, gamified approaches, and engaging narratives that make the educational content more enjoyable and memorable. Cluster 2 balances fun and educational content. This corresponds to the principle of Enjoyable Education, which stresses the importance of making educational content both engaging and enjoyable.
- Immersive experience (PR3): create gameplay mechanics and features that immerse players in the game world, enhancing the overall experience. Creating gameplay mechanics and features that immerse players in the game world enhances the overall experience. By focusing on details such as realistic graphics, immersive audio, intuitive controls, and captivating storytelling, players can become fully absorbed in the game, fostering a sense of presence and enhancing their emotional connection with the game environment. Cluster 3 relies on creating immersive and challenging games. This maps to the principle of immersive experience, focusing on enhancing player immersion through engaging gameplay mechanics and features.
- Player engagement (PR4): designing games that cater to specific player preferences and learning needs increases player engagement. This can involve providing customizable options, diverse gameplay styles, and adaptive mechanisms that allow players to approach the game in ways that align with their interests and individual learning styles. Cluster 4 highlights its focus on tailoring games to individual learning styles and preferences. This aligns with the principle of player engagement, which involves designing games that cater to specific player interests and learning needs.
- Motivate continuous learning (PR4): incorporating gameplay mechanics and features that motivate players to continue playing and learning is crucial. This can be achieved through elements such as rewards, achievements, leaderboards, progression systems, and unlocking new content. By offering tangible incentives and a sense of progression, players are encouraged to keep playing, exploring, and learning. Cluster 5 prioritizes player experience and problem-solving. This corresponds to the principle of motivate continuous learning, emphasizing the importance of encouraging ongoing engagement and learning through game mechanics.
- Effective learning outcomes (PR5): designing a game experience that supports effective learning outcomes involves aligning educational objectives with gameplay elements. This includes incorporating interactive challenges, problem-solving scenarios, and knowledge- or application-based tasks that promote active learning and meaningful engagement. Cluster 6 is related to unique or innovative elements such as augmented reality. This aligns with the principle of effective learning outcomes, which involves aligning educational objectives with gameplay elements to support meaningful learning not only playing for fun.
- Positive behaviors (PR6): provide a gaming experience while encouraging desirable behaviors. This involves rewards for completing educational tasks or promoting prosocial behaviors within the game context. By fostering positive behaviors and attitudes, games can have a broader impact on players’ lives beyond the game itself. Cluster 7 contains fun and engaging games with educational content. This maps to the principle of positive behaviors, focusing on incorporating positive reinforcement and promoting desirable behaviors through game design.

## Discussion

### Principal Findings

The field of serious games has emerged as an effective digital solution for traditional education, training, and behavior change. However, designing effective DSGs that achieve their intended outcomes is a complex process that requires a thorough understanding of the design frameworks used in both entertainment games (DEGDFWs) and serious games (DSGDFWs). The purpose of this scoping review was to investigate the design frameworks used in both types of games and explore how they can inform the design and development of serious games.

The review involved identification and analysis of the recurring steps that appeared across the selected game design frameworks. The synthesis of the reviewed frameworks involved several steps. First, the frameworks were analyzed to identify key elements, structures, and processes relevant to game development. Common components and phases across them were then extracted, such as game mechanics, player engagement, learning outcomes, and iterative design. Next, frameworks with overlapping concepts were grouped together, highlighting similarities in approach, such as those focusing on player behavior and personality, game mechanics and dynamics, or the integration of educational content.

This section discusses the key findings of the scoping review by presenting a visualization of the 4-phase design. It must be noted that this is not a design framework proposal but a conceptual and visual summary of the reviewed design space for DSG design. In addition, a set with guiding design principles are mapped on each proposed design phase to better inform the future design initiatives.

The synthesis of common steps into the 4 design phases baseline directly cross-references insights from the literature review and cluster analysis, which emphasized the structured concepts essential for effective educational game design. For instance, the importance of aligning game mechanics with educational objectives, aligning with the conceptualization phase identified in the current analysis. Similarly, the previous sections discussed the necessity of detailed planning in preproduction, echoing the importance of narrative and educational goals in game development.

Although each framework was unique in terms of its specific components, several key steps were consistently noted. These steps included conceptualization—where the initial idea or game concept is formed; preproduction planning—involving the detailed planning of game mechanics, narrative, and educational goals; prototyping iterations—where initial versions of the game are developed, tested, and refined; and evaluation—a process of assessing the game’s effectiveness, either through playtesting or measuring educational outcomes after its deployment in production. By recognizing these common steps, the analysis distilled the frameworks into a more abstract structure, which pointed to an underlying shared design flow as a 4 design phases baseline. This synthesis was further refined by grouping these steps into 4 overarching phases: exploration, design, development, and assessment.

In essence, the commonalities and dependencies identified in the previous sections of the paper directly align with key considerations in each phase of the 4-phase design baseline flow: initial exploration should be guided by an understanding of who the players are; design should integrate engagement, learning, and outcomes; development should be iterative, incorporating user input; and assessment should be continuous to enable refinement. The reliance on entertainment models for engagement shows the importance of player-centered research on motivations and interests in the initial exploration while still scoping learning goals. With many frameworks leveraging entertainment concepts such as narrative and challenge for enjoyment, the design phase should focus on seamlessly integrating both fun and pedagogy into cohesive mechanics. The adaptation and customization approaches demonstrate the need for iterative, user-centered development that continually gathers feedback to refine serious games to be more responsive to learners. The goal of continuous improvement highlighted in the clusters requires embedding assessment throughout the process to evaluate learning and track engagement, enabling real-time identification of issues through analytics.

Furthermore, this section presents some detected gaps as highlights for future research. By understanding these gaps, researchers and designers can focus on addressing them for the goal to enhance the effectiveness of serious games in real practice.

### DSGDFW Baseline With 4 Design Phases

#### Overview

As a result of the analysis performed during the literature review, several high-level phases that were common across both entertainment and serious games, despite differences in their intended outcomes, were revealed. For example, key phases such as conceptualization, preproduction planning, prototyping iterations, and evaluation processes were noted consistently. These common phases pointed to a shared design thinking structure, even when specific tools and techniques differed between entertainment and instructional contexts.

**Figure 3:**
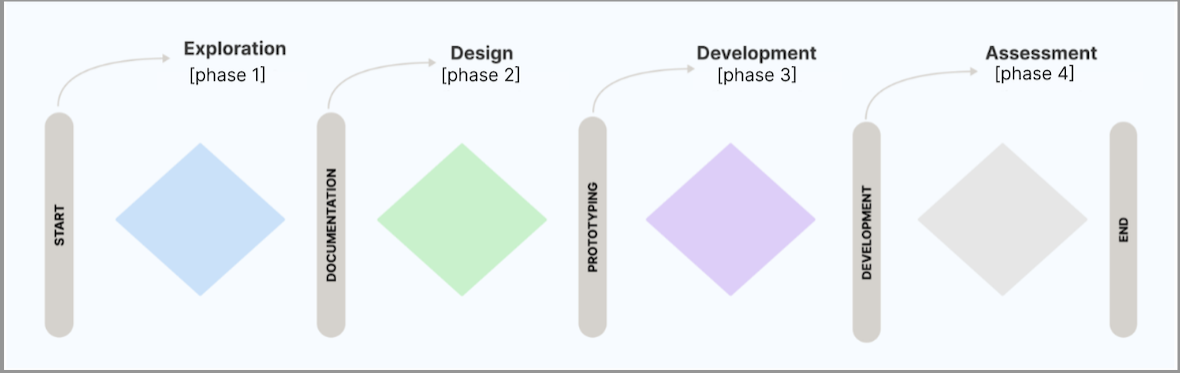
Overview of the 4-design phase.

These common phases inform an integrated design flow encapsulating exploration, design, development, and assessment, which have been synthesized in this section, summarized in Figure 3. While specifics within each phase need tailoring or change depending on each paper included in the review, the macrolevel sequencing of the design process itself provides an intuitive skeleton. This enabled simplicity in the baseline visualization focused on connecting sequential design stages shared across frameworks. Moving forward, further layers of complexity can be added within phases. The visualization therefore aims to provide clarity to serious game creators on the core high-level workflow derived from common practices identified through analysis.

The exploration phase sets the stage for integration by interweaving learning principles and content considerations with gameplay factors from the outset, thereby ensuring the entertainment and education elements synergize.

The identified commonalities and dependencies principles such as emphasis on player experience, game mechanics consideration, challenge tailoring, and immersive experience, from previous sections, informed this first phase. During this phase, understanding player motivations and preferences is central. Both DEG and DSG frameworks stress the need for designers to focus on creating enjoyable and engaging player experiences. The game mechanics must be conceptualized here, as they influence challenge levels, engagement, and the player’s interaction with the game’s content.

#### Exploration Phase

In the exploration phase, the key elements are defining the main idea, intended audience, learning goals, and identifying user profiles and risks. The Conceptual Model for Serious Games provides a framework for designing serious games by defining the key design elements of the game, such as goals, challenges, rewards, and feedback. For instance, GAMED methodology focuses on identifying user needs and preferences by considering the game’s content, structure, and user interface, while digital game–based learning—instructional design (DGBL-ID) model helps to determine the learning objectives and target users of the proposed game by following a structured approach to designing and developing educational games. This phase is important as it lays the foundation for the entire serious game development process.

**Figure 4:**
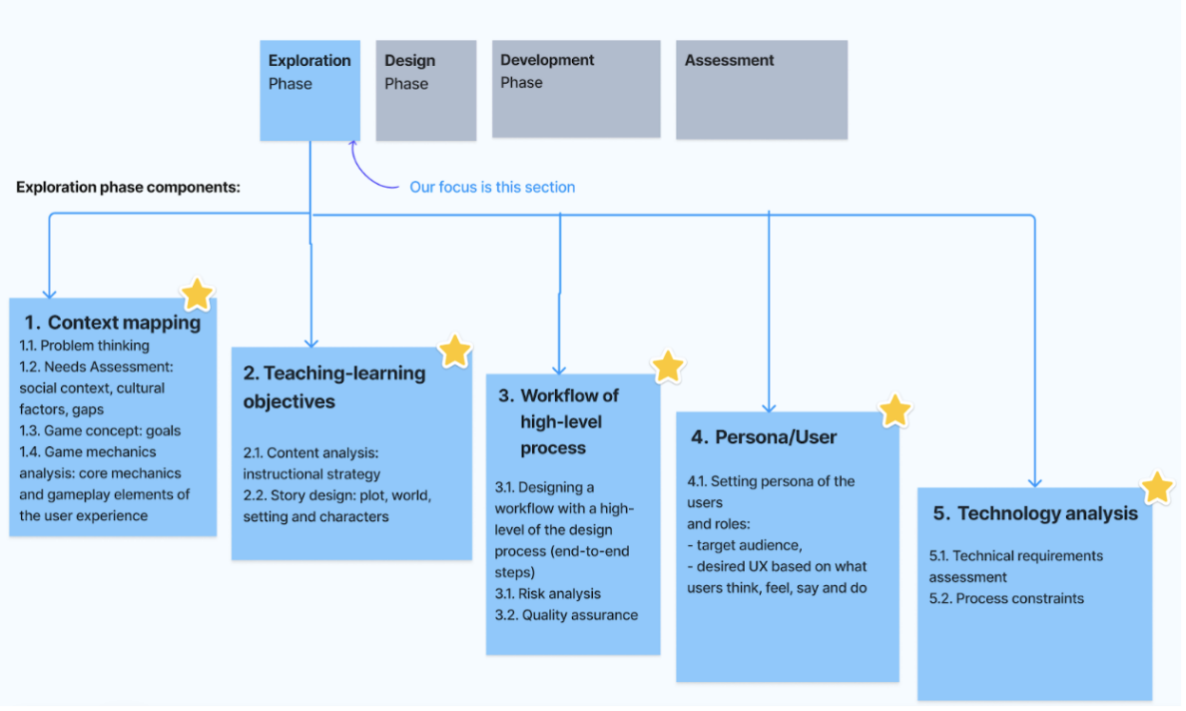
Exploration phase. UX: user experience.

The exploration phase of serious game design requires careful consideration of various components as shown in Figure 4. Context mapping involves problem thinking, identifying the social context, cultural factors, and gaps through a needs assessment, while the game concept is established by defining the game goals. Game mechanics analysis is used to analyze the core mechanics and gameplay elements of the user experience, while teaching-learning objectives are set by conducting a content analysis to determine the instructional strategy. Story design is developed to establish the plot, world, setting, and characters. Workflow design is essential for developing games systematically and efficiently, and involves risk analysis and quality assurance. Setting the persona of the users and roles is critical to identifying the target audience and designing the desired user experience through empathy map modeling. Finally, technology analysis involves assessing technical requirements and process constraints to ensure that the game is developed within the necessary technological limitations.

When designing serious games during the exploration phase, it is important to follow these guidelines to ensure thoughtful consideration of various aspects. The EG1-6 guidelines are correlated with the principles and commonalities (eg, DEG-C1-7, DSG-C1-7, and PR1-6) outlined in the previous sections (Textbox 6).

**EG1-6 guidelines.**

- EXP-G1: understand the problem and educational needs—focus on identifying the learning gaps and instructional challenges to be addressed, ensuring the game design supports effective learning outcomes (PR5 and PR6). Take into account player experience (DEG-C1) and feedback (DSG-C4) to create a customized solution that meets both educational and motivational needs (DSG-C5).
- EXP-G2: define game goals and overall concept—set clear learning objectives aligned with the educational content, incorporating instructional design principles (DSG-C2) and pedagogical modeling (DSG-C5). Ensure the balance between educational and entertainment content (PR2) to create enjoyable learning experiences (DEG-C1 and DSG-C1).
- EXP-G3: outline core gameplay mechanics and dynamics—design adaptive and immersive gameplay mechanics (PR1 and PR3) that integrate educational content (DSG-C1). Tailor the challenges to player abilities (DEG-C2) and ensure mechanics are interconnected with esthetics and narrative elements (DEG-C3) to promote continuous engagement (PR4).
- EXP-G4: determine instructional strategies for teaching players—analyze and design the content delivery approach by blending entertainment game elements with instructional strategies. Focus on gameplay mechanics that reinforce learning objectives (PR5), and integrate psychological aspects such as motivation and memory (DSG-C6) to enhance player engagement and retention (PR4).
- EXP-G5: develop an engaging narrative and world—create a compelling story (DEG-C7) and immersive game environment (DSG-C3) that supports the educational goals. The narrative should guide players through their learning journey, ensuring the plot and game tasks align with instructional outcomes (PR3 and PR2), while maintaining the focus on motivation and enjoyment (PR2).
- EXP-G6: conduct risk analysis and establish quality standards—assess potential risks and ensure the game adheres to quality assurance standards. Play testing (DEG-C4) and iteration (DEG-C5) are key for refining the game design to maintain balance between difficulty, engagement, and educational content (PR1 and PR5).
- EXP-G7: identify and customize for the target audience—research and define the characteristics of the target learners, including their knowledge levels, learning styles, and motivational drivers (DSG-C5 and DSG-C6). Customize the game design to meet these needs by offering adaptive learning paths (PR4) and immersive experiences tailored to player preferences (DEG-C6).

Overall, the exploration phase takes a broad focus on laying the strategic groundwork through investigative research and early concept ideation to identify well-grounded opportunities where purposeful games can deliver substantial educational value. The phase delivers clarity on the needs, goals, possibilities, and priorities to focus the global initiative.

The design phase serves as the critical foundation that informs all subsequent stages of serious game creation by merging educational and entertainment elements into a unified vision. It is during design that learning goals get translated into concrete gameplay mechanics, dynamic challenges, and a narrative interweaving obstacle progression with instructional scaffolding. Established pedagogical frameworks should provide guidance to map activities supporting knowledge construction, mastery through application, evaluative feedback, and metacognitive reflection.

The interconnectivity of game components, importance of play testing, immersive experience, and effective learning outcomes commonalities and principles are linked to this design phase. The narrative, esthetics, and game mechanics must work together harmoniously, aligning with both DEG and DSG frameworks. The iterative process begins here, allowing the game design to evolve based on user feedback, enhancing the player experience. Incorporating immersive elements, such as realistic graphics or compelling storylines, is essential. DSG frameworks emphasize aligning gameplay with educational objectives, meaning that the mechanics designed must not only be engaging but also facilitate learning.

#### Design Phase

In the design phase, the key elements are designing the game environment, mechanics, scenarios, objects, and architecture, and capturing and defining the framework of the game process. For instance, the Conceptual Model for Serious Games provides a framework for designing serious games by defining the key design elements of the game. The DGBL-ID model focuses on creating the graphical user interface and designing the game’s fantasy and story context. The RETAIN model emphasizes the transfer of knowledge and repetition of use in designing the game’s fantasy and story context. This phase is important as it encompasses the creative and artistic elements of the serious game, shaping its visual, audio, and interactive aspects. Well-designed game elements are essential in creating an engaging, immersive, and enjoyable experience for players, which can enhance their motivation, learning outcomes, and overall experience with the serious game.

**Figure 5:**
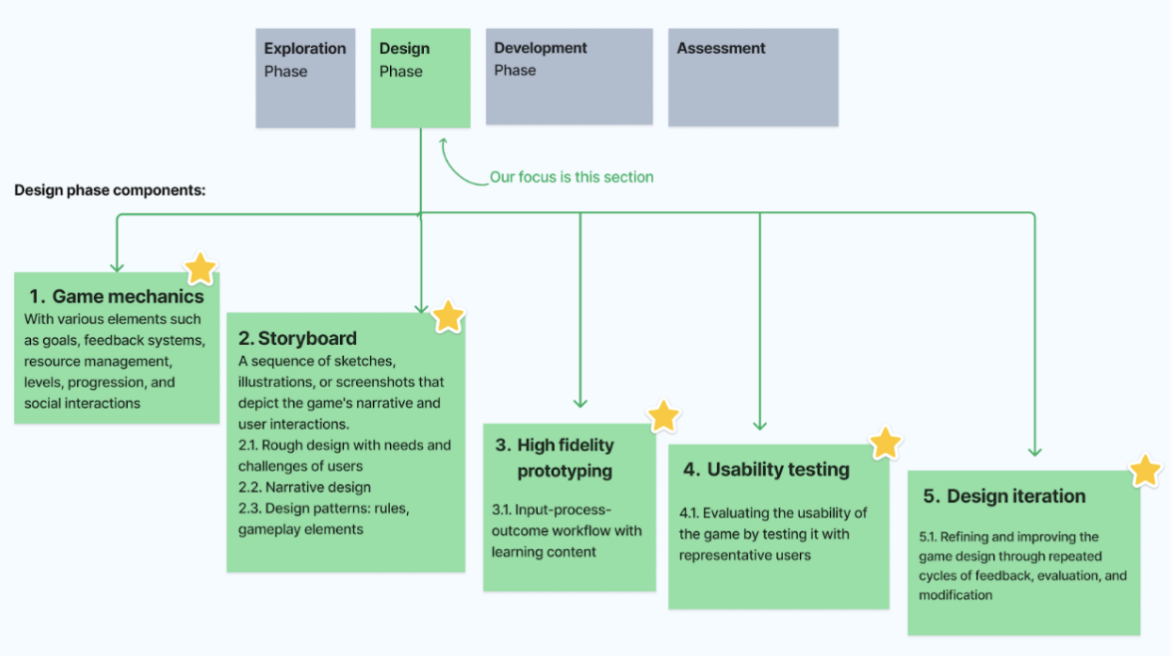
Design phase.

The design phase of serious game design consists of several components as shown in Figure 5. The first component involves the actual design of game mechanics, which has been agreed upon in the previous phase of analysis. The second component is the creation of a storyboard for the serious game solution, which includes a rough design with the needs and challenges of users, a narrative design, and agreed design patterns such as rules and gameplay elements. The third component involves creating a high-fidelity prototype, which includes an input-process-outcome workflow with learning content. The fourth component is usability testing, which involves testing the usability of the serious game solution with users. The final component is DSG design iteration before development, which allows for feedback and improvements to be made before the development phase begins.

The guidelines listed are important in the design phase as they provide a foundation for decision-making. The Dg1-5 guidelines are aligned with the principles and commonalities (eg, DEG-C1-7, DSG-C1-7, and PR1-6) from earlier sections, ensuring that the design phase is informed by established frameworks (Textbox 7).

**Dg1-5 guidelines.**

- DES-G1: align mechanics with analysis—map game mechanics (DEG-C2) to player engagement (DEG-C1 and PR4) and learning objectives (DSG-C1, DSG-C5, and PR5), ensuring that challenges (PR1) and positive behaviors (PR6) are tailored to players’ cognitive abilities and motivations (DEG-C6 and DSG-C6). Instructional design principles (DSG-C2) should guide this alignment to ensure the educational content is effectively delivered.
- DES-G2: create a comprehensive storyboard—develop a rich narrative (DEG-C7) that integrates educational content with storytelling (PR2) and immersive elements (DEG-C3, DSG-C3, and PR3). The storyboard should map out how the player’s progression and learning activities (DEG-C2 and DSG-C1) become essential for advancing through challenges (PR1) and achieving effective learning outcomes (PR5).
- DES-G3: build high-fidelity prototypes—build prototypes that focus on immersive gameplay mechanics (DEG-C3, DSG-C3, and PR3) and collaboration features (DSG-C7), ensuring they are realistic and engaging (PR2 and PR4). These prototypes should test the integration of educational content with learning loops and performance feedback (DSG-C1 and PR5), fostering early insights into player engagement and behaviors (PR4 and PR6).
- DES-G4: test usability with users in the early phase—conduct usability testing with target learners to gather feedback (DEG-C4 and DSG-C4) on engagement, usability, and learning effectiveness (PR5). This early feedback loop helps refine the alignment of game mechanics with player motivations and cognitive aspects (DEG-C6, DSG-C6, and PR4), ensuring the gameplay supports effective learning outcomes (PR5).
- DES-G5: iterate design before development—iterate on the game design (DEG-C5) based on playtesting (DEG-C4 and DSG-C4) and feedback on engagement, challenge tailoring (PR1), and positive behaviors (PR6). This iterative process ensures that the game’s educational content and mechanics are refined to enhance both player experience and learning results (PR5).

Overall, effective serious games arise not from simply layering a thin game on top of educational content, but from baking deep learning principles into design thinking itself. So the blend of engagement and outcomes begins here by thoughtfully transitioning high concepts around pedagogy and theories into an emergent unified practice.

#### Development Phase

In the development phase, the key element is developing the game using programming software and graphic editing tools. For example, the DGBL-ID model provides a framework for developing educational games by following a structured approach to designing and developing the game. This phase is very important as it brings the game to life by incorporating the designed elements into a functional game. Efficient development processes, coding, and deployment are essential in ensuring that the game functions as intended and meets the design requirements.

The development phase considers elements from the mapped commonalities and dependencies principles such as play testing in the real built game where the improvements area of the games can be deepened as it was in the prototyping phase with limited clickable actions. The importance of storytelling is another key principle that guides the coding of game mechanics, ensuring a smooth, end-to-end flow in narrative sequences and also influences the development of graphics, audio, and video assets. Collaboration and communication are elements that interrelate all 4 design phases, but their importance is even higher in this phase in which designers from the previous 2 phases of exploration and design should concisely share the strategy with deep details to both game front- and backend engineers.

The development phase is critical for serious games because it determines how well the conceptual design gets translated into an actual playable, educational experience. With so many technical components to coordinate, it is easy for the learning goals to get lost in the coding efforts and asset creation. That is why the highest focus during development should remain on manifesting the learning theories and instructional strategies that were outlined earlier.

Keeping the target learners at the center, developers need to constantly question how each new mechanic and gameplay element reinforces the intended skill-building instead of solely crafting an entertaining experience. The educational content should drive the creation of media assets to correctly illustrate target knowledge instead of generic placeholders. Programming adaptive levels requires understanding how to model evolving mastery. Iterative playtesting verifies that embedded assessments provide useful diagnostic data to tell how well learners are acquiring expertise.

In essence, the biggest risk is cleanly designed learning principles not making an intact transition into the implemented product. By emphasizing the instructional elements as the true measures of quality assurance over graphics polish or clever moderately fun interactions lacking constructive pedagogical alignment, serious game developers keep advancing toward the vision of fusing engagement with substantive outcomes originally conceived. The highest focus stays on the target users achieving growth.

On top of the development phase components, artificial intelligence (AI) can play a very important role in supporting serious game personalization. AI machine learning algorithms can adapt game content for learners’ motivation. AI techniques, such as procedural content generation, can automate the creation of game content, levels, and scenarios. AI-powered natural language processing techniques can enable communication and interaction with game characters through speech or text. This opens up possibilities for language learning, dialogue-based decision-making, or simulated conversations within serious games [109,110].

**Figure 6:**
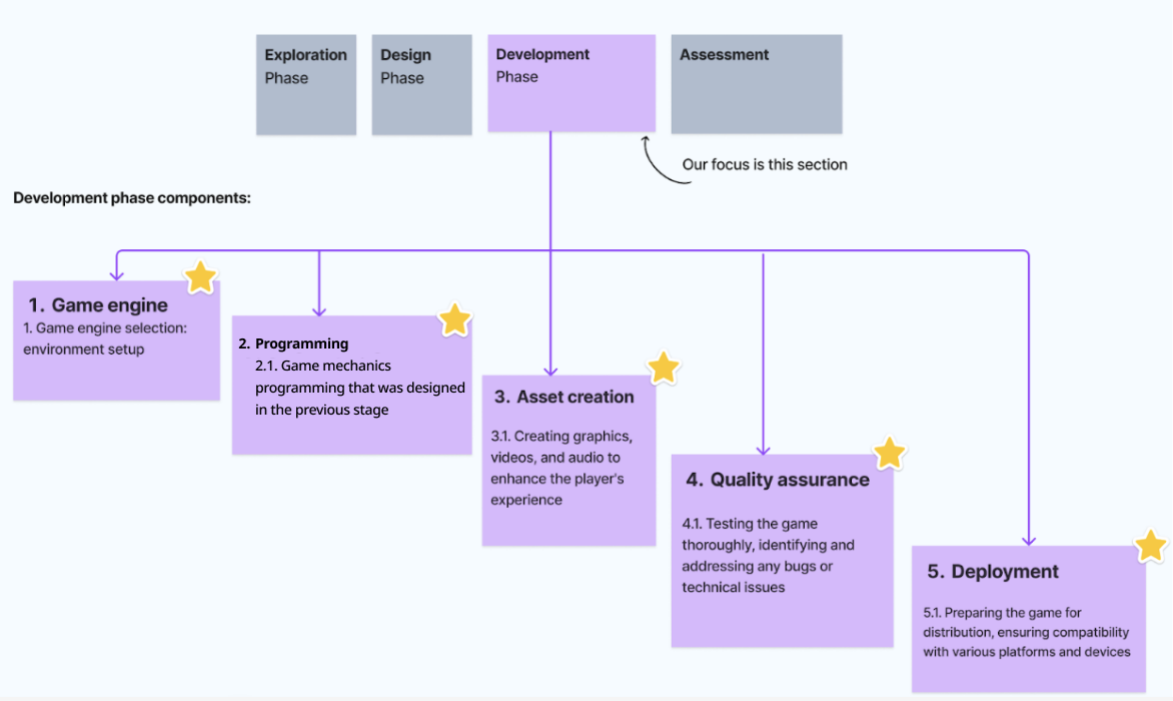
Development phase.

The components for the development phase of serious game design represent the technical implementation of the game as shown in Figure 6. Game engine selection involves choosing an appropriate game engine for the development of the game, and setting up the environment for development. Game mechanics programming involves coding the game mechanics that were designed in the previous phase. Asset creation involves the creation of graphics, videos, and audio that are required for the game, by building the game’s characters, environments, and animations, as well as creating sound effects and background music. Quality assurance ensures that the game functions correctly, is free of bugs and glitches, and meets all necessary standards. Deployment and distribution involves making the game available for users to access and play.

In the development phase, designers are encouraged to incorporate some guidelines alongside the specified components. The development guidelines (DEV-G) are aligned with the principles and commonalities (eg, DEG-C1-7, DSG-C1-7, and PR1-6) from earlier sections (Textbox 8).

**DEV-G guidelines.**

- DEV-G1: choose an appropriate game engine for development—select a game engine that supports embedding assessment analytics (DSG-C5), allowing the integration of mechanics tailored to player skills (PR1) and immersive educational experiences (PR5). The engine should support both seamless gameplay (DEG-C3) and educational outcomes (DSG-C1).
- DEV-G2: set up the development environment—create a modular environment that facilitates iterative refinement (DEG-C5 and DSG-C2) and supports adaptive algorithms for customizing challenges (PR1). This setup helps balance player experience (DEG-C1) and learning content (DSG-C5), allowing continual adjustments based on learner feedback (PR2 and PR4).
- DEV-G3: code the game mechanics based on the design—develop game mechanics that integrate learning objectives (DSG-C1 and DSG-C5) and player experience elements (DEG-C1 and DEG-C2), ensuring the mechanics are tailored to challenge players progressively (PR1) while delivering educational content in an engaging way (PR2). Gameplay mechanics should also reinforce immersive experiences (PR3).
- DEV-G4: build game characters, environments, and animations—design characters and environments that provide an immersive experience (DEG-C3 and DSG-C3), ensuring the game world is engaging and supports storytelling (DEG-C7 and PR5). Adaptive gameplay should respond to player emotions and motivations (DEG-C6 and DSG-C6), fostering engagement and promoting continuous learning (PR4).
- DEV-G5: develop sound effects and background music—use sound and music to enhance immersion (PR3 and DEG-C3) and motivate positive behaviors (PR6). Well-designed audio cues can support both storytelling (DEG-C7) and the reinforcement of educational tasks (DSG-C5), contributing to an overall engaging player experience (DEG-C1 and DSG-C4).
- DEV-G6: conduct quality assurance to ensure bug-free and glitch-free gameplay—thorough testing (DEG-C4 and DSG-C4) should be conducted with target users to identify usability issues affecting engagement (PR4) and ensure the gameplay supports continuous learning (PR2). Iteration based on playtesting ensures refined mechanics (PR1) and better player experience (DEG-C1).
- DEV-G7: meet all necessary standards for the game—ensure the game complies with relevant industry and educational standards (DSG-C2 and DSG-C5), integrating structured instructional design principles (DSG-C2) and mechanics that align with effective learning outcomes (PR5). The game must also maintain player engagement (DEG-C1) while meeting technical standards for performance and interactivity (DEG-C3).
- DEV-G8: deploy and distribute the game for user access and play—ensure that the final game deployment supports seamless access (DEG-C3), providing a smooth user experience (DEG-C1) and aligning with learning objectives (DSG-C1 and DSG-C5). After launch, continue gathering feedback for further iterations (DEG-C5) to maintain engagement and foster learning (PR2 and PR4).

Overall, the purpose of the development phase in serious game design is to effectively translate the learning theories and instructional strategies from the conceptual stages into a functional, playable experience that meaningfully impacts the target learners. It is when the foundations in educational content and gameplay principles finally converge into integrated software centering on the knowledge and skill acquisition goals that propelled this endeavor. By prioritizing learner needs through iterative testing and refinement, serious game developers fulfill the promise of leveraging engagement to achieve substantive growth and performance gains—the core vision that galvanized the exploration, design, and now development workflow. The end goal remains focused on players completing the experience with measurable, demonstrable educational gains at scale.

#### Assessment Phase

The assessment phase is the fourth phase in this visualization of the design space for serious games with the objective to show evidence on whether the game is achieving the pedagogical goals that justified its development in the first place. Compared to entertainment games where success is measured purely by player enjoyment and engagement, serious games necessitate more complex evaluation based on learning principles.

The assessment phase is informed by commonalities and principles such as catering the game experience to player-learners’ motivations for long-term satisfaction, effective learning outcome, player engagement and positive behaviors. This highlights the need to ensure the final product aligns with various player types and objectives. The game’s mechanics, narrative, educational content, and user experience are measured against the initial design goals, ensuring that both entertainment value and learning outcomes are achieved.

Both quantitative and qualitative data gathered from embedded assessments, gameplay analytics, standardized tests, interviews, surveys, and other instruments should tie back to the original learning objectives, content integration blueprints, and target competencies identified during the exploration phase. Statistical analysis combined with input from expert educators helps determine alignment and efficacy.

Assessment is not a one-time event, either. The promise of serious games lies in their capacity to be continually refined and adapted at scale based on learner performance patterns. So systematic assessment should feed back into development iterations that improve instructional strategy integrations for existing players while also informing subsequent game designs.

In the assessment phase, the key elements are measuring the quality of the game and testing the prototype for effectiveness and usability by the target users. For example, GAMED methodology focuses on evaluating the game’s quality by indicators such as acceptability, clarity, effectiveness, engageability, enjoyability, interactivity, localizability, rewardability, simplicity, transformativeness, and usability. The six-dimensional framework examines the various components of mobile game–based learning to provide learners with a mobile or ubiquitous learning experience. The DGBL-ID model emphasizes testing the prototype and real game for effectiveness and usability by the target users.

This phase is required in assessing the effectiveness of the serious game in achieving its intended learning goals and identifying areas for improvement. Even in this phase, AI can be supportive by analyzing large volumes of player data generated during gameplay to extract valuable insights. By examining player behavior, performance patterns, and learning outcomes, AI algorithms can provide valuable feedback to game designers and educators. These insights can inform the iterative design process, allowing for continuous improvement and optimization of serious games [110].

**Figure 7:**
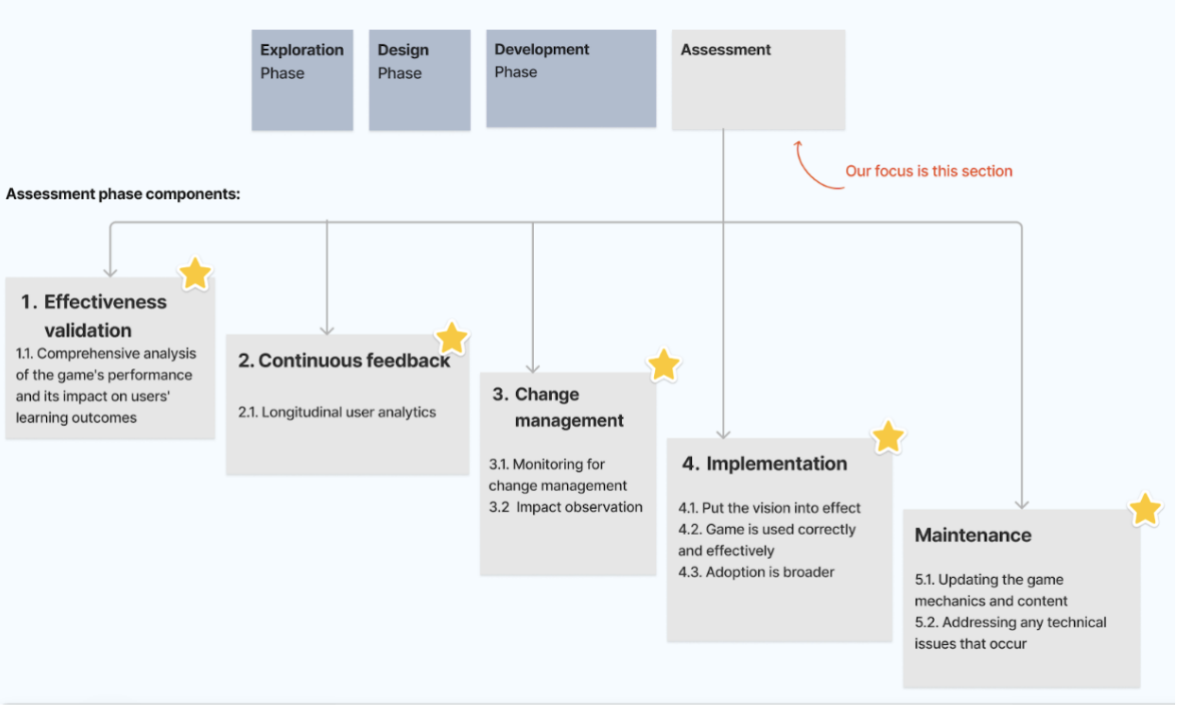
Assessment phase.

The components of the assessment phase, as shown in Figure 7, outline the steps necessary to achieve the general goals and objectives initially established. The effectiveness validation is the first component of this phase, where designers conduct a comprehensive analysis of the game’s performance and its impact on users’ learning outcomes. The continuous user feedback collection is another vital component, which involves gathering feedback from users through various channels to identify areas for improvement and enhance the user experience. The third component is monitoring for change management, where designers keep track of the game’s performance and the broader context to adapt the game’s design to better meet changing user needs. Implementation is the fourth component, where designers ensure that the game is used correctly and effectively by providing training to users or incorporating it into a broader training program. Finally, maintenance is the fifth component, which involves regularly updating the game’s content, fixing bugs, and addressing any technical issues to ensure that it remains effective in achieving its intended goals and continues to provide a positive user experience.

During the assessment phase, designers should consider the guidelines alongside the specified components. These guidelines (ASM-G1-G6) are mapped to the relevant principles, or commonalities (eg, DEG-C1-7, DSG-C1-7, and PR1-6) from the previous sections (Textbox 9).

Overall, the assessment phase should use empirical data from a pedagogical lens to validate and continuously enhance learning outcomes. In essence, the validation needed for serious games to proliferate relies on demonstrating tangible learning gains.

While assessment is codified as a key overarching phase, evaluation and user feedback mechanisms should be embedded across the design and development cycle. Effective serious game design necessitates agile, continual review of educational effectiveness beyond entertainment engagement. Frequent playtesting research and qualitative user input must directly shape iterative refinement of instructional content and game mechanics.

**Assessment guidelines (ASM-G) G1-G6.**

- ASM-G1: achieve general goals and objectives initially set up—align the game design with player experience goals (DEG-C1) and learning objectives (DSG-C1), ensuring the gameplay mechanics challenge players appropriately (DEG-C2 and PR1). By focusing on effective learning outcomes (PR5), the game should support both entertainment and educational objectives.
- ASM-G2: validate effectiveness through comprehensive analysis—leverage gameplay metrics to assess the interconnectivity of components (DEG-C3) and ensure immersive experiences (PR3) that align with instructional design principles (DSG-C2). Conduct analysis using embedded assessments (DSG) and data mining to validate both engagement and learning outcomes (PR4 and PR5).
- ASM-G3: collect continuous user feedback for improvement—use playtesting (DEG-C4) to gather qualitative feedback on player experience (DEG-C1) and gameplay mechanics (DEG-C2). Ensure continuous iterations based on player feedback (DEG-C5) to improve motivation and learning engagement (PR4). Collect and refine feedback to align with educational content (DSG-C1 and PR2).
- ASM-G4: monitor for change management and adapt design accordingly—ensure adaptive algorithms align with player skill levels (PR1), and adjust immersive elements (DEG-C3, PR3) to maintain engagement. Refine the game mechanics and pedagogical strategies (DSG-C5 and PR5) based on continuous monitoring and feedback from players (PR4).
- ASM-G5: ensure correct and effective use through implementation—develop analytics dashboards to track player progression and learning outcomes (DSG-C5 and PR5). Align game mechanics with cognitive aspects (DSG-C6) to ensure that gameplay not only engages players (DEG-C1) but also reinforces desired behaviors (PR6) through positive feedback mechanisms.
- ASM-G6: maintain games through regular updates, bug fixes, and addressing technical issues—regularly update game mechanics (DEG-C2) and immersive elements (PR3) to align with advancing technology and changing player preferences. Ensure the continued collection of feedback (DEG-C4) and maintain player engagement through iterative improvements (DEG-C5 and PR4).

Assessment seems like a final phase but it is strongly related to previous phases of design (usability testing) and development (quality testing), as a continuous goal during this user-centered design process flow. During the initial exploration phase, rapid crowdsourced needs validation surveys help establish user criteria for the subsequent evaluation stages. Usability testing in the design phase prioritizes educational obstacles alongside entertainment engagement early on. Quality assurance procedures throughout the development gather user perspectives on improving learning outcomes alongside gameplay.

Ongoing analysis of interaction telemetry, knowledge assessments, qualitative feedback, and field pedagogy insights provides continuous signals to refine serious game effectiveness. By embedding ongoing reciprocal user input opportunities, the design space phases foster natural critical review cycles rather than one-way static reporting.

Ultimately, quality user research interactions allow designers and developers to dynamically adapt game elements on the fly to better achieve target learning goals. Assessment interwoven across phases provides that tight, iterative development feedback loop for optimization.

### Detected Gaps in DSGDFWs

Serious game development frameworks have made significant strides in addressing various aspects of the field. However, there remain gaps that could be addressed to further enhance the effectiveness of serious game development. One such gap is the consideration of cultural diversity. Many existing frameworks may not fully take into account the impact of cultural differences in learning styles or preferences. Some researchers highlight the importance of cultural differences in game performance with pointers that might inspire how this gap can be reduced. Future frameworks could explore ways to incorporate cultural diversity considerations into serious game design to ensure that games are inclusive and appealing to a broader range of learners [111].

Another area that requires more attention in serious game development frameworks is ethical considerations. With the increasing use of serious games in educational settings, it is crucial to address issues such as data privacy, user consent, and potential biases. Ethical considerations related to serious game development, including the responsible handling of user data and ensuring unbiased representation of diverse groups, should be explicitly addressed in frameworks to ensure the ethical use of serious games in educational contexts. One pointer for this gap would be for designers to ensure that ethical considerations are at the forefront of their decision-making process [112].

Sustainability is another gap in many serious game development frameworks. While current frameworks often focus on the development of individual games, there is limited consideration of the long-term impact of serious games and their sustainability over time. DSG developers could incorporate approaches for creating serious games that have a longer life span, can be updated or scaled, and remain relevant and effective in the face of technological advancements and changing educational needs.

Interdisciplinary collaboration is recognized as an influential aspect of serious game development, but existing frameworks may not provide detailed guidance on how to facilitate this effectively. Collaboration between different stakeholders, such as game designers, educators, and researchers, is essential for creating high-quality serious games. Future frameworks could include more explicit guidance on fostering interdisciplinary collaboration and communication, including strategies for effective team dynamics and communication among diverse stakeholders throughout the serious game development process.

In addition, some serious game development frameworks may not explicitly incorporate empathic design principles, even though they emphasize user-centered design. Empathic design, which involves understanding the user’s needs, emotions, and experiences, is crucial in creating meaningful serious games. Addressing the gap of empathic design principles in serious game design involves prioritizing the understanding of lecturers and players’ emotions, needs, and experiences. Designers could explicitly integrate empathic design principles, recognizing the importance of designing serious games that truly resonate with the needs and experiences of the lecturers-players-learners [113].

Overall, while DSGDFW have made significant progress, there remain gaps that could be addressed to further enhance the field. Consideration of cultural diversity, ethical considerations, sustainability, interdisciplinary collaboration, and empathic design are areas that could benefit from more explicit inclusion in serious game development frameworks, ensuring that serious games are effective, inclusive, and ethically responsible educational tools.

### Conclusions

This study explored DEGs and DSGs with the aim of identifying the approaches used in the frameworks, their commonalities and gaps, as well as the fundamental design elements and phases that can inform better design strategies for future DSGs. This scoping review has identified key findings that can inform the future design and development of serious games. By leveraging the design frameworks used in both DEGs and DSGs, designers and developers can create effective games that meet their intended outcomes and expectations of learners. DEGs design elements such as adaptive challenge levels based on player’s skill level or performance [51,83], cultivation of continuous curiosity through exploration, provision of clear game objectives for confusion reduction and motivation increase [79], creation of deep engaging experience shared in collaboration and competition between players [76], and immediate reward tailored to player achievements [83] are inspirational for serious game design.

This review of established entertainment and serious game design frameworks discerned common phases in development processes, alongside recurring interdependencies between engagement models and instructional components. Synthesizing framework research revealed the design space visualization in the 4 design phases outlined as one of the outcomes in this paper—exploration, design, development, and assessment—as a baseline for DSG development, with each phase playing an essential role in the overall success of the game. The exploration phase involves defining the main idea, intended audience, learning goals, and identifying user profiles and risks. This phase sets the foundation for the entire serious game development process and allows for informed decision-making and strategic planning. The design phase focuses on designing the game environment, mechanics, scenarios, objects, and architecture, and capturing and defining the framework of the game process. Well-designed game elements are essential in creating an engaging, immersive, and enjoyable experience for players. The development phase involves developing the prototype of the game using programming software and graphic editing tools, ensuring that the game functions as intended and meets the design requirements. Finally, the assessment phase involves measuring the quality of the game and testing the prototype for effectiveness and usability by the target users. The resulting visualization aims to give clarity, so developers can efficiently craft serious games across domains based on research-driven best practices. This design process underscores that the lines separating games and learning are far more connected at their core.

The contribution of this review that other papers have not addressed in serious game design frameworks lies in several aspects. First, it explores the dependencies between DEGDFWs and DSGDFWs, providing insights into the approaches applied in both frameworks and identifying design elements that can inform future better design strategies for serious games. This broader perspective allows designers and developers to leverage the design frameworks used in entertainment games to create more effective serious games that meet their intended outcomes and learners’ expectations. Second, the study proposes 4 key design phases (exploration, design, development, and assessment) that serve as a baseline for serious game development, providing a structured foundation for decision-making and strategic planning and execution. These phases address the need for a holistic approach to serious game design and emphasize the importance of considering various aspects throughout the end-to-end buildout process.

The guiding design heuristics derived from the principles of dependency analysis can be easily mapped to the generic 4-design-phase flow. The exploration phase should focus on assessing learning needs, player preferences, and target outcomes grounded in principles of enjoyment, engagement, and behavioral change. The design phase aims to translate this knowledge into an experiential vision, seamlessly integrating education and immersion through balanced challenges and incentivized mastery. The development phase uses continuous feedback and analytical tracking to iterate real-time adaptations, supporting personalized progress with positive reinforcement. Finally, the assessment phase prioritizes measurable learning impacts verified empirically while gathering player perspectives to improve engagement quality by recalibrating enjoyment and difficulty. Across all stages, identified principles guide purposeful experiential configurations, inform tradeoffs, set data collection priorities, direct prototyping efforts, and shape evaluation standards for optimizing serious gameplay efficacy.

Finally, the study highlights the significance of some gaps such as empathic design approach in serious game design, recognizing the potential of understanding users’ needs and expectations to create more engaging and effective games. Empathic design thinking can revolutionize serious game design in fields such as general education and health care training, resulting in games that are tailored to the unique needs, preferences, and motivations of the target users, which are lecturers and learners.

In summary, the expansive literature scoping, multimodal analysis, theoretical model contribution, actionable guidelines for practice, and overarching conceptual connections orient this paper to deliver unique significance advancing the field. The serious game design frameworks have shown promise in a variety of fields, including education, health care, and training. Overall, although this study makes a valuable contribution to the field of DSGs by proposing a well-defined design foundation, continuous research is necessary to fully realize the potential of creating serious games that effectively address the evolving needs of future generations.

### Future Work

There are several future trends in serious game design frameworks that might emerge from recent research. One trend is the use of data analytics and AI to personalize serious game experiences for individual users, based on their preferences, abilities, and health status. This can improve the effectiveness of serious games by tailoring them to the needs of each user. Finally, there is a need for application of empathic design thinking approach for a greater collaboration between game designers and end users (lecturers and learners) that serious games are designed and evaluated in a way that is scientifically rigorous and meets their needs.

In future research, we might explore the potential of AI mixed with empathic design thinking approaches for serious game design and development. For example, an AI-based game design framework that uses facial recognition and machine learning to personalize the game content and difficulty level based on the player’s emotions and cognitive effort.

## Appendix

Multimedia Appendix 1 PRISMA (Preferred Reporting Items for Systematic Reviews and Meta-Analyses) 2020 checklist.

Multimedia Appendix 2 Full search strategy for included databases.

Multimedia Appendix 3 Purposes of digital serious game design frameworks.

Multimedia Appendix 4 Results with papers with digital entertainment game design frameworks (n=16).

Multimedia Appendix 5 Results with papers with digital serious game design frameworks (n=31).

## Abbreviations

AI: artificial intelligence
DEG: digital entertainment game
DEGDFW: digital entertainment game design framework
DGBL-ID: digital game–based learning—instructional design
DSG: digital serious game
DSGDFW: digital serious game design framework
PRISMA: Preferred Reporting Items for Systematic Reviews and Meta-Analyses
