# Supplementary material for: Identifying Key Principles and Commonalities in Digital Serious Game Design Frameworks: Scoping Review
Source: JMIR Serious Games. 2025 Mar 5;13:e54075. doi: 10.2196/54075 (PMC11923477; doi:10.2196/54075)
Supplement: Multimedia Appendix 2 [file games_v13i1e54075_app2.pdf]

Supporting information

Table S1. Full search strategy for included databases.

Database	Search strategy
ACM	((serious games * "design" OR "frameworks" OR "guidelines"))<br>((games * "design" OR "frameworks" OR "guidelines"))
Scopus	TITLE-ABS-KEY (((serious games * "design" OR "frameworks" OR "guidelines")))<br>(((games * "design" OR "frameworks" OR "guidelines")))
Springer	((serious games * "design" OR "frameworks" OR "guidelines"))<br>((games * "design" OR "frameworks" OR "guidelines"))
IEEE	((serious games * "design" OR "frameworks" OR "guidelines")))<br>(((games * "design" OR "frameworks" OR "guidelines"))
Elsevier	(serious games * "design" OR "frameworks" OR "guidelines"))<br>(((games * "design" OR "frameworks" OR "guidelines"))
JMIR	((serious games * "design" OR "frameworks" OR "guidelines")))<br>(((games * "design" OR "frameworks" OR "guidelines"))
SAGE	((serious games * "design" OR "frameworks" OR "guidelines")))<br>(((games * "design" OR "frameworks" OR "guidelines"))
