# Supplementary figures and images for: Identifying Key Principles and Commonalities in Digital Serious Game Design Frameworks: Scoping Review
Source: JMIR Serious Games. 2025 Mar 5;13:e54075. doi: 10.2196/54075 (PMC11923477; doi:10.2196/54075)

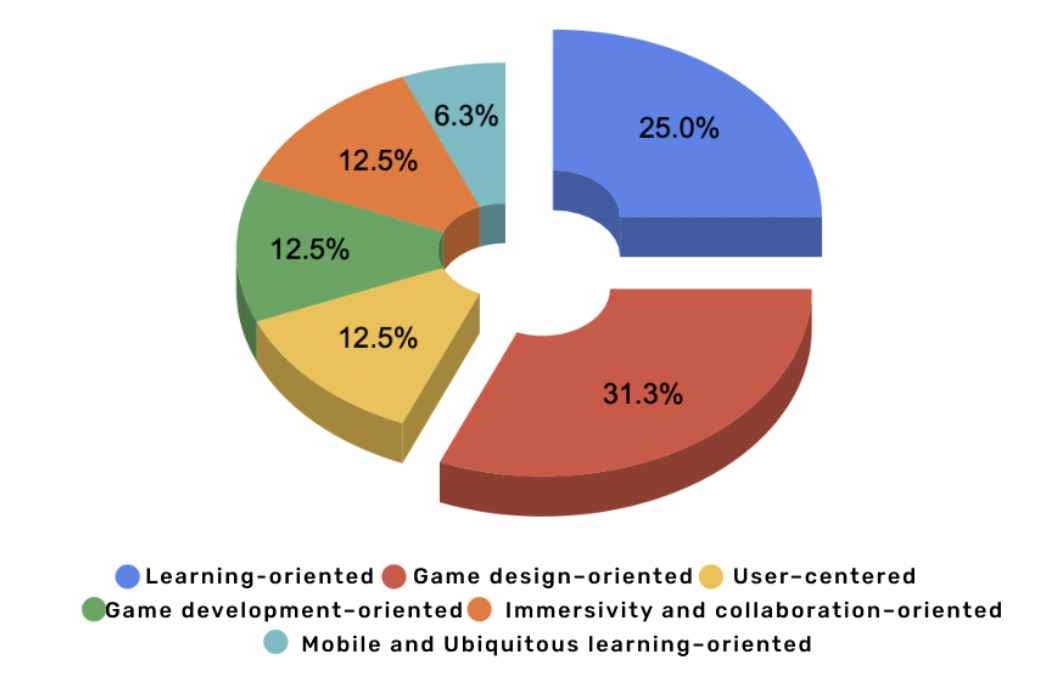

Supplement: Multimedia Appendix 3 [file games_v13i1e54075_app3.png]
