# Supplementary material for: Identifying Key Principles and Commonalities in Digital Serious Game Design Frameworks: Scoping Review
Source: JMIR Serious Games. 2025 Mar 5;13:e54075. doi: 10.2196/54075 (PMC11923477; doi:10.2196/54075)
Supplement: Multimedia Appendix 4 [file games_v13i1e54075_app4.pdf]

Table 1. Results table of studies and the reviewed Digital Entertainment Game Design Workflows (DEGDFW) (N=16).

ID code	DEGDFW	Description
EG1	The Bartle Taxonomy [45]	Categorizes players into four types (achievers, killers, explorers, and socialisers) based on their motivations. The framework can be used to tailor the design of a game to appeal to specific player types. Players have different motivations and goals when playing a game, and understanding these motivations can help game designers create more engaging and rewarding game experiences.
EG2	The Four Keys to Fun [46]	It can be used to understand the different types of fun that can be achieved through game mechanics and to ensure that the game is challenging and engaging for players. It identifies four key elements: easy, hard, people, and serious fun.
EG3	The Engines of Play [47]	Relates to Big Five personality profiling tools that can be applied in game design to understand how different player personalities may affect the level of immersion in the game. It assesses players based on five dimensions: Openness to Experience, Conscientiousness, Agreeableness, Extraversion and Neuroticism.
EG4	The Player Involvement Model [48]	Used to assess play experiences by analyzing metrics such as players' attentional resources and the cognitive load of games. It helps to understand the various levels of immersion and involvement that players may experience during gameplay.
EG5	MDA (Mechanics, Dynamics, Aesthetics) [44]	It can be used to analyze and understand game components. It has a long history of application in game design and can be used to understand how different mechanics, dynamics, and aesthetics

		contribute to the overall player experience.
EG6	The Layered Tetrad [54]	Combines three models, including Formal, Dramatic, and Dynamic Elements; Elemental Tetrad; and MDA, to understand the various elements that contribute to the overall player experience. The framework considers mechanics, dynamics, aesthetics, and story as key components of player experience.
EG7	Design, Dynamics, Experience (DDE) [40]	Consists of Design, Dynamics, and Experience. The framework emphasizes creating a meaningful experience for the player by considering their emotional and psychological responses during gameplay. The goal is to create games that are fun and provide impactful experiences for the players.
EG8	Elemental Tetrad [52]	It comprises four components: mechanics, aesthetics, story, and technology. The components are interconnected with each other in game design, with mechanics and aesthetics being directly linked. Even though story and technology may be less visible to players, they are still vital components in game design, and are interconnected with every other component.
EG9	MTDA+N Narratives Framework [53]	Includes five elements: Mechanics, Technology, Dynamics, Aesthetics, and a Narratives Framework.
EG10	Integrated Framework for Game Design [41]	Proposes a six-step formal framework to guide game designers through different design stages. The steps include Theme, Main Aesthetic, Core Mechanic, Subordinate Aesthetics, Adjust Difficulty, and Validate and Iterate. It suggests that the designer should approach a general Theme with a specific discourse, identify the Main Aesthetic that communicates the discourse, and iteratively Validate and Iterate the design.
EG11	The 5-part Model	Comprises five properties: Representation, Tokens, Resources,

	[51]	Goals, and Decisions, which can be used to create and diagnose game mechanics. Tokens and Resources are subsets of Representation that the player can control directly or 'spend' and 'gain', respectively. The Goals include Immediate, Local, and Global goals, while Decisions focus on the interactive aspect of the game and give players the power to use or not to use a mechanic, and to manage resources spent while using it.
EG12	Risk/Reward Model, (difficulty balancing) [50]	Suggests that game difficulty can be adjusted through factors like locked difficulty, dare, skill level, and time. Designers can use cost as risk and gain as reward to fine-tune the difficulty of a game for an engaging experience.
EG13	Game Design Patterns Model [49]	Integrates game design with design patterns. It includes formal, dramatic, and dynamic elements.
EG14	Game design Framework [42]	Integrates a game design framework with design patterns, specifically the state, strategy, prototype, and observer patterns. The framework is divided into formal, dramatic, and dynamic elements, which can be implemented using different design patterns. Formal elements can use state, strategy, and observer patterns, dramatic elements can use creational patterns such as prototype, and dynamic elements can use state, strategy, observer, and potentially prototype patterns depending on the nature of the game emergence and story.
EG15	Game design workshop [55]	Covers various aspects of game design, from the role of the game designer to selling oneself and ideas to the game industry. The workshop includes the structure of games, working with formal and dramatic elements, system dynamics, conceptualization, prototyping, digital prototyping, play testing, functionality, completeness, and balance, fun and accessibility, team structures,

		stages of development, and understanding the game industry. The design document is also discussed as an essential part of the game development process.
EG16	Game Element and Mechanic (GEM) Framework [43]	Analyzes the different elements and mechanics of games. The Game Element model identifies three layers, focusing on exploration and narrative, achievement and validation, and dexterity and skill. The Game Mechanic model underlines efficacy, activism, socialization, and organization. The analysis shows correlations between different elements and mechanics, and highlights which ones are preferred or avoided by players.

References:

40. Walk W, Görlich D, Barrett M. Design, dynamics, experience (DDE): an advancement of the MDA framework for game design. In: Blatz M, Korn O, Amato A, Walk W, editors. Game Dynamics: Best Practices in Procedural and Dynamic Game Content Generation. Cham, Switzerland. Springer; 2017:27-45. doi:10.1007/978-3-319-53088-8_3

41. Mora-Zamora R, Brenes-Villalobos E. Integrated framework for game design. In: Proceedings of the IX Latin American Conference on Human Computer Interaction. 2019. Presented at: CLIHC '19; September 30-October 4, 2019:1-6; Panama City, PA. URL: https://dl.acm.org/doi/10.1145/3358961.3358984 [doi: 10.1145/3358961.3358984]

42. Barakat NH. A framework for integrating software design patterns with game design framework. In: Proceedings of the 8th International Conference on Software and Information Engineering. 2019. Presented at: ICSIE '19; April 9-12, 2019:47-50; Cairo, Egypt. URL: https://dl.acm.org/doi/10.1145/3328833.3328871 [doi: 10.1145/3328833.3328871]

43. Ferro LS. The game element and mechanic (GEM) framework: a structural approach for implementing game elements and mechanics into game experiences. Entertain Comput. Jan 2021;36:100375. [doi: 10.1016/j.entcom.2020.100375]

44. Hunicke R, LeBlanc M, Zubek R. MDA: A formal approach to game design and game research. In: Proceedings of the 2004 AAAI Workshop on Challenges in Game AI. Washington, DC. Association for the Advancement of Artificial Intelligence; 2004.

45. Bartle RA. Hearts, clubs, diamonds, spades: players who suit MUDs. J Virtual Environ. 1996;1:1-16. [FREE Full text]
46. Lazzaro N. Why we play games: four keys to more emotion without story. XEODesign. 2004. URL: https://gamemodworkshop.com/readings/xeodesign_whyweplaygames.pdf [accessed 2024-04-29]
47. Zimmerman E. The engines of play. In: Salen K, Zimmerman E, editors. The Game Design Reader: A Rules of Play Anthology. Cambridge, MA. MIT Press; 2006:633-645.
48. Bartle R. Designing Virtual Worlds. Berkeley, CA. New Riders; 2004.
49. Björk S, Zagal JP. Game design and role-playing games. In: Zagal JP, Deterding S, editors. Role-Playing Game Studies: Transmedia Foundations. New York, NY. Routledge; 2018:323-336.
50. Wang C, Huang L. A systematic review of serious games for collaborative learning: theoretical framework, game mechanic and efficiency assessment. Int J Emerg Technol Learn. Mar 30, 2021;16(06):88. [doi: 10.3991/ijet.v16i06.18495]
51. Costikyan G. I have no words and I must design: toward a critical vocabulary for games. In: Proceedings of the 2002 Conference on Computer Games and Digital Cultures Conference. 2002. Presented at: CGDC '02; June 6-8, 2002:1-25; Tampere, Finland. URL: https://dl.digra.org/index.php/dl/article/view/29/29 [doi: 10.4324/9781003140184-10]
52. Schell J. The Art of Game Design: A Book of Lenses. New York, NY. Morgan Kaufmann; 2008.
53. Monu K, Ralph P. Beyond gamification: implications of purposeful games for the information systems discipline. arXiv. Preprint posted online August 2, 2013. [FREE Full text]
54. Gibson J. Introduction to Game Design, Prototyping and Development: From Concept to Playable Game - with Unity and C-sharp. Pearson Education Inc; 2014;430-480. ISBN:978-0321933164
55. Fullerton R. Game Design Workshop: A Playcentric Approach to Creating Innovative Games. Boca Raton, FL. CRC Press; 2018.
