# Supplementary material for: Identifying Key Principles and Commonalities in Digital Serious Game Design Frameworks: Scoping Review
Source: JMIR Serious Games. 2025 Mar 5;13:e54075. doi: 10.2196/54075 (PMC11923477; doi:10.2196/54075)
Supplement: Multimedia Appendix 5 [file games_v13i1e54075_app5.pdf]

Table 2. Results table of studies and the reviewed Digital Serious Game Design Workflows (DSGDFW) (N=31).

ID code	DSGDFW	Description
SG1	Activist-Casual framework [75]	Outlines principles for developing engaging serious games with a positive message and meaningful choices. It suggests starting with simple game mechanics and gradually increasing complexity, adjusting difficulty based on performance, and offering personalization. Ethical monetization, iterative prototyping, and play testing are also emphasized.
SG2	Educational games design framework [93]	Focuses on game design elements such as usability, multimodality, and fun, as well as pedagogy based on Bloom's Taxonomy outcomes (knowledge, comprehension, and application) and learning content modeling through scaffolding.
SG3	Design, Play, Experience Framework [95]	Consists of three layers: educational content for learning outcomes, storytelling, and gameplay (actions and rules), and also considers the user experience, including entertainment.
SG4	Conceptual framework [91]	Combines instructional content with the capability to learn, and includes game attributes for learning activities and reflection, as well as game mechanics and game genre for achieving learning goals.
SG5	Four Dimensional Framework [92]	Has four dimensions: context-game scenarios, representation with visuals and characters, learner attributes, and pedagogy based on problem-based learning.
SG6	Triadic game design evaluation framework [97]	Based on the triadic game design philosophy, which emphasizes the importance of considering the three worlds of reality, meaning, and play in game design and evaluation.
SG7	RETAIN framework [96]	An evaluation framework for educational games that focuses on the immersion of academic content within the game's fantasy and story context, the transfer of knowledge, and the repetition of usage.

SG8	Computational Puzzle Design (CPD) framework [70]	A tool for designing games that teach computational thinking. It consists of three aspects: the components of CT, specific activities, and gameplay elements. The games aim to teach players to solve problems through programming using non-coding methods like drag-and-drop tools.
SG9	Serious educational games framework (SG-LMI) [94]	Includes five components: identity (personalization and identification with the game), immersion (engagement with the game world), interactivity (active participation in the game), increasing complexity (progressive difficulty of game tasks), and informed teaching and instructional design.
SG10	FRACH framework [67]	Aims to facilitate collaboration and communication between archaeologists, pedagogues, and designers in creating serious games. It promotes the exchange of artifacts to facilitate communication and design and links learning goals, immersivity, and collaboration to enhance group learning and understanding.
SG11	Augmented intelligence framework [73]	Includes identifying the cognitive behavior and factors that the game aims to improve, determining the intended users, designing the game scenario, selecting the devices to be used, identifying the necessary modules from the augmented intelligence technology, and determining the game's input mechanisms.
SG12	Lu Lu framework [78]	Consists of two dimensions: lusory and ludic. The lusory dimension includes the goal of the game and the means to achieve it, the efficiency of achieving the goal, and the complexity of the system. The ludic dimension includes game mechanics, a story, technology, and aesthetics.
SG13	Learning Mechanics - Game Mechanics [63]	Suggests that effective serious games should integrate educational content into gameplay through the use of learning mechanics and game mechanics. Learning mechanics involve guidance, experimentation, and assessment, while game mechanics include role play, levels, and feedback.

SG14	Digital Game Based Learning – Instructional Design (DGBL – ID) Model [64]	Includes five phases: analysis, design, development, quality assurance, and implementation and evaluation. In the process, the learning objectives and target users are identified, the graphical user interface is created, a prototype is developed and tested in the quality assurance phase.
SG15	Immersive Educational Games Model [65]	Comprises elements of instructional design, gameplay, immersion, and serious game theories to outline the key considerations in creating engaging educational gameplay.
SG16	Design, Dynamics, Experience (DDE) framework [40]	A new approach to game design that emphasizes the player's perspective and experience as a journey, incorporating the concept of Player-Subject. It considers narrative design as an integral part of game design, advocating for an iterative production process, and it enhances the understanding of the player's experience.
SG17	MECONESIS methodology [84]	A Human Interaction Computer (HCI) approach for designing serious games. It involves analyzing user needs and requirements in an educational context, and incorporating a learning style. It identifies aspects in game mechanics, such as the most appropriate device, continuous feedback, challenges, scoring system, and learning levels.
SG18	Framework with game design, learning content modeling, and pedagogy [85]	Combines game design, learning content modeling, and pedagogy with a multimodal perspective.
SG19	Methodology based on graphic notation and interactive narrative [86]	Based on graphic notation and interactive narrative, integrates transversal aspects and uses visual representations to facilitate communication between team members during the development of a serious game.
SG20	Methodology based on cognitive-behavior techniques [87]	Focuses on cognitive-behavior techniques and has a psychological approach that allows students to experience different emotions while playing.

SG21	EMERGO methodology [88]	A methodology and toolkit for developing serious games to enhance the cognitive skills of secondary school students.
SG22	ATMSG framework based on Activity Theory [89]	A conceptual model that supports the systematic representation of educational games based on pedagogical objectives using Activity Theory.
SG23	Methodology based on problem-based learning [90]	Facilitates the integration of educational content using problem-based learning. It involves creating games that present real-world problems to encourage the player to work through these challenges using critical thinking and problem-solving skills.
SG24	Six-dimensional framework, in reviewed paper [68]	Includes Spatiotemporal, Collaboration/Social, Session, Personalization, Data security and privacy, and Pedagogy. These dimensions are related to gaming characteristics, learning strategies, personalisation, pedagogy, and data security and privacy issues in existing mobile-games based learning solutions.
SG25	Baseline content-centric framework [69]	Based on more layers that imply understanding differences between game design, game design thinking and game development. Briefly, this framework presents the interconnection between game mechanics, prototyping, framing, vertical design, prioritizing backlogs and play testing in the end.
SG26	GAMED framework [71]	Comprises four phases and a dozen processes, and provides a structured approach for overcoming the complexity of DEG development. The quality of a DEG is measured by a set of indicators including acceptability, challengeability, clarity, effectiveness, engageability, enjoyability, interactivity, localizability, rewardability, simplicity, transformativeness, and usability.
SG27	User-centered design (UCD) methodology [72]	Focuses on the needs and preferences of the user throughout the game development process. It involves techniques such as playtesting, usability testing, and gathering initial user experiences through surveys and focus groups.

SG28	Collaborative Learning Game framework [76]	Has a strong focus on game mechanics with six elements: space, objectives/states, actions, rules/goals, skills and chance.
SG29	Conceptual Model for Serious Games [77]	A four-phase model for game development consisting of analysis, design, development, and evaluation. During analysis, the main idea and user profiles are identified. In design, the game environment, mechanics, and architecture are created. Development involves coding and documentation, and the evaluation phase includes continuous testing and assessment of learning goals and game quality.
SG30	Adaptive Learning Game Design (ALGAE) Model [79]	Facilitates improved learning outcomes. It proposes a new workflow consisting of five key components: Learner, Action, Conflict, Cooperation, and Instructional strategies. These components interact with each other and with the game engine to create a dynamic and engaging learning experience that adapts to the learner's needs and preferences.
SG31	Intervention Mapping Framework [80]	Contains six steps: exploring the problem, defining change objectives, selecting behavior change techniques, designing the intervention, guaranteeing implementation, and evaluation. Each step provides a specific task and methods that guide the subsequent step, but is time-consuming for designers.

References:

40. Walk W, Görlich D, Barrett M. Design, dynamics, experience (DDE): an advancement of the MDA framework for game design. In: Blatz M, Korn O, Amato A, Walk W, editors. Game Dynamics: Best Practices in Procedural and Dynamic Game Content Generation. Cham, Switzerland. Springer; 2017:27-45.

63. Proulx JN, Romero M, Arnab S. Learning mechanics and game mechanics under the perspective of self-determination theory to foster motivation in digital game based learning. Simul Gaming. Nov 02, 2016;48(1):81-97. [doi: 10.1177/1046878116674399]

64. Mat Zin NA, Jaafar A, Wong SY. Digital game-based learning (DGBL) model and development methodology for teaching history. WSEAS Trans Comput. 2009;8:323-333. [FREE Full text] [doi: 10.1163/9789004719118_030]

65. Baker J, Wanick V, Asiri M, Wills G, Ranchhod A. Immersion and narrative design in educational games across cultures. In: Ma M, Oliveira MF, Petersen S, Hauge JB, editors. Serious Games and Edutainment Applications. Volume II. Cham, Switzerland. Springer; 2017:605-621.

67. Andreoli R, Corolla A, Faggiano A, Malandrino D, Pirozzi D, Ranaldi M, et al. A framework to design, develop, and evaluate immersive and collaborative serious games in cultural heritage. J Comput Cult Herit. Dec 07, 2017;11(1):1-22. [doi: 10.1145/3064644]

68. Giannakas F, Kambourakis G, Papasalouros A, Gritzalis S. A critical review of 13 years of mobile game-based learning. Educ Tech Res Dev. Dec 12, 2017;66(2):341-384. [doi: 10.1007/s11423-017-9552-z]

69. Tsikinas S, Xinogalos S. Designing effective serious games for people with intellectual disabilities. In: Proceedings of the 2018 IEEE Global Engineering Education Conference. 2018. Presented at: EDUCON '18; April 17-20, 2018:1-6; Santa Cruz de Tenerife, Spain. URL: https://ieeexplore.ieee.org/document/8363467 [doi: 10.1109/educon.2018.8363467]

70. Jiang X, Harteveld C, Huang X, Fung AY. The computational puzzle design framework: a design guide for games teaching computational thinking. In: Proceedings of the 14th International Conference on the Foundations of Digital Games. 2019. Presented at: FDG '19; August 26-30, 2019:1-11; San Luis Obispo, CA. URL: https://dl.acm.org/doi/10.1145/3337722. 3337768 [doi: 10.1145/3337722.3337768]

71. Aslan S, Balci O. GAMED: digital educational game development methodology. Simulation. Mar 02, 2015;91(4):307-319. [doi: 10.1177/0037549715572673]

72. Moosa AM, Al-Maadeed N, Saleh M, Al-Maadeed SA, Aljaam JM. Designing a mobile serious game for raising awareness of diabetic children. IEEE Access. 2020;8:222876-222889. [doi: 10.1109/access.2020.3043840]

73. Golestan S, Mahmoudi-Nejad A, Moradi H. A framework for easier designs: augmented intelligence in serious games for cognitive development. IEEE Consumer Electron Mag. Jan 2019;8(1):19-24. [doi: 10.1109/mce.2018.2867970] 93. Daylamani-Zad D, Angelides MC, Agius H. Lu-Lu: a framework for collaborative decision making games. Decis Support Syst. May 2016;85:49-61. [doi: 10.1016/j.dss.2016.02.011]

75. King D. Integrating serious and casual game design approaches: a framework for activist-casual game design. In: Proceedings of the 16th International Conference on the Foundations of Digital Games. 2021. Presented at: FDG '21; August 3-6, 2021:1-9; Montreal, QC. URL: https://dl.acm.org/doi/10.1145/3472538.3472568 [doi: 10.1145/3472538.3472568]

76. Wang C, Huang L. A systematic review of serious games for collaborative learning: theoretical framework, game mechanic and efficiency assessment. Int J Emerg Technol Learn. Mar 30, 2021;16(06):88. [doi: 10.3991/ijet.v16i06.18495]

77. Avila Pesantez D, Delgadillo R, Rivera L. Proposal of a Conceptual Model for Serious Games Design: A Case

Study in Children With Learning Disabilities. IEEE Access. 2019;7:127480-127489. doi:10.1109/ACCESS.2019.2951380

78. Daylamani-Zad D, Angelides MC, Agius H. Lu-Lu: a framework for collaborative decision making games. Decis Support Syst. May 2016;85:49-61. [doi: 10.1016/j.dss.2016.02.011]

79. Lamb RL, Annetta L, Firestone J, Etopio E. A meta-analysis with examination of moderators of student cognition, affect, and learning outcomes while using serious educational games, serious games, and simulations. Comput Human Behav. Mar 2018;80:158-167. [doi: 10.1016/j.chb.2017.10.040]

80. Pouls BP, Bekker CL, van Dulmen S, Vriezekolk JE, van den Bemt BJ. A serious puzzle game to enhance adherence to antirheumatic drugs in patients with rheumatoid arthritis: systematic development using intervention mapping. JMIR Serious Games. Feb 18, 2022;10(1):e31570. [FREE Full text] [doi: 10.2196/31570] [Medline: 35179510]

84. Cano S, Arteaga JM, Collazos CA, Gonzalez CS, Zapata S. Toward a methodology for serious games design for children with auditory impairments. IEEE Latin America Transactions. 2016;14(5):2511-2521. https://api.semanticscholar.org/CorpusID:1822949

85. Ibrahim R, Jaafar A. Educational games design framework: Combination of game design, pedagogy and content modeling. Proceedings of Electrical Engineering and Informatics, 2009. ICEEI'09. International Conference on; 2009;293-298. https://api.semanticscholar.org/CorpusID:15247176

86. de Lope RP, Arcos JRL, Medina-Medina N, Paderewski P, Gutiérrez-Vela F. Design methodology for educational games based on graphical notations: Designing Urano. Entertainment Computing. 2017;23:42-54. doi: 10.1016/j.entcom.2016.08.005

87. Szczesna A, Tomaszek M, Wieteska A. The methodology of designing serious games for children and adolescents focused on psychological goals. Proceedings of Information Technologies in Biomedicine. Springer; 2012. p. 151-162. doi: 10.1007/978-3-642-31196-3_24

88. Nadolski RJ, Hummel HG, Van Den Brink HJ, Hoefakker RE, Slootmaker A, Kurvers HJ, et al. EMERGO: A methodology and toolkit for developing serious games in higher education. Proceedings of Simulation & Gaming. 2008;39(3):338-352. https://api.semanticscholar.org/CorpusID:62220776

89. Carvalho MB, Bellotti F, Berta R, De Gloria A, Sedano CI, Hauge JB, et al. An activity theory-based model for serious games analysis and conceptual design. Proceedings of Computers & Education. 2015;166-181. https://api.semanticscholar.org/CorpusID:4142693

90. Barbosa AF, Pereira PN, Dias JA, Silva FG. A new methodology of design and development of serious games. Int J Comput Games Technol. 2014;2014:1-12. doi: 10.1155/2014/817167
91. Yusoff A, Crowder R, Gilbert L, Wills G. A conceptual framework for serious games. Proceedings of the Ninth IEEE International Conference on Advanced Learning Technologies. 2009;15-17. doi: 10.1109/ICALT.2009.19

92. De Freitas S, Jarvis S. A framework for developing serious games to meet learner needs. Proceedings of the Interservice/Industry Training, Simulation and Education Conference, Florida; 2006;50. https://api.semanticscholar.org/CorpusID:14239688

93. Ibrahim R, Jaafar A. Educational games (EG) design framework: Combination of game design, pedagogy and content modeling. Proceedings of the International Conference on Electrical Engineering and Informatics; 2009;1:293-298. doi: 10.1109/ICEEI.2009.5254771

94. Annetta LA. The "I's" have it: A framework for serious educational game design. Rev Gen Psychol. 2010;14(2):105-112. doi: 10.1037/a0018985

95. Winn BM. The design, play, and experience framework. In: Handbook of Research on Effective Electronic Gaming in Education. 2008;5497:1010-1024. doi: 10.4018/978-1-59904-808-6.ch058

96. Gunter GA, Kenny RF, Vick EH. Taking educational games seriously: using the RETAIN model to design endogenous fantasy into standalone educational games. Educ Tech Res Dev. Oct 16, 2007;56(5-6):511-537. [doi: 10.1007/S11423-007-9073-2]

97. Harteveld C. Triadic game evaluation: a framework for assessing games with a serious purpose. In: Proceedings of the 2010 Design and Engineering of Game-like Virtual and Multimodal Environments Workshop. 2010. Presented at: DEGVME '10; June 20, 2010:5-11; Berlin, Germany. URL: https://www.researchgate.net/publication/
